# Increased PD-L1 expression and IL-6 secretion characterize human lung tumor-derived perivascular-like cells that promote vascular leakage in a perfusable microvasculature model

**DOI:** 10.1038/s41598-017-09928-1

**Published:** 2017-09-06

**Authors:** Colette A. Bichsel, Limei Wang, Laurène Froment, Sabina Berezowska, Stefan Müller, Patrick Dorn, Thomas M. Marti, Ren-Wang Peng, Thomas Geiser, Ralph A. Schmid, Olivier T. Guenat, Sean R. R. Hall

**Affiliations:** 1 ARTORG Organs-on-Chip Technologies, University of Bern, Bern, Switzerland; 2 Department of Pulmonary Medicine, Inselspital, Bern University Hospital, Bern, Switzerland; 3 Department of BioMedical Research, University of Bern, Bern, Switzerland; 4 Division of General Thoracic Surgery, Inselspital, Bern University Hospital, Bern, Switzerland; 5 Department of Pathology, University of Bern, Bern, Switzerland; 6 Flow cytometry core facility, Department of BioMedical Research, University of Bern, Bern, Switzerland; 7 Present Address: Vascular Biology Program, Boston Children’s Hospital, Boston, MA USA

## Abstract

Pericytes represent important support cells surrounding microvessels found in solid organs. Emerging evidence points to their involvement in tumor progression and metastasis. Although reported to be present in the human lung, their specific presence and functional orientation within the tumor microenvironment in non-small cell lung cancer (NSCLC) has not yet been adequately studied. Using a multiparameter approach, we prospectively identified, sorted and expanded mesenchymal cells from human primary NSCLC samples based on co-expression of CD73 and CD90 while lacking hematopoietic and endothelial lineage markers (CD45, CD31, CD14 and Gly-A) and the epithelial marker EpCAM. Compared to their normal counterpart, tumor-derived Lineage-EpCAM-CD73+CD90+ cells showed enhanced expression of the immunosuppressive ligand PD-L1, a higher constitutive secretion of IL-6 and increased basal αSMA levels. In an *in vitro* model of 3D microvessels, both tumor-derived and matched normal Lineage-EpCAM-CD73+CD90+ cells supported the assembly of perfusable vessels. However, tumor-derived Lineage-EpCAM-CD73+CD90+ cells led to the formation of vessels with significantly increased permeability. Together, our data show that perivascular-like cells present in NSCLC retain functional abnormalities *in vitro*. Perivascular-like cells as an eventual target in NSCLC warrants further investigation.

## Introduction

Fibroblasts are traditionally synonymous with the source of activated stromal cells found in solid tumors^1^. Evidence from animal models points to tumor-derived transforming growth factor beta 1 (TGF-β1) as a key mediator in converting fibroblasts in the tumor stroma to alpha-smooth muscle actin (αSMA)-expressing myofibroblasts termed cancer-associated myofibroblasts or CAFs^2^. It is this activated stroma that is now recognized as having a central role in tumor initiation, growth and progression, as well as resistance in several solid tumor types^3, 4^. However, the origin of the myofibroblast remains a challenge, as a number of different stromal or mesenchymal cells have been identified that may provide a viable pool of precursor cells contributing to CAFs ranging from resident fibroblasts to extrapulmonary mesenchymal stromal cells^5, 6^.

Since activated tumor stroma resembles fibrotic tissue in that there is involvement of myofibroblasts and excessive wound healing^1^, findings from fibrotic tissue may reveal related mechanisms in cancer progression. Recent data examining fibrosis in solid organs such as the liver and kidney points to an important role of resident perivascular cells or pericytes as a potential source of myofibroblast precursor cells^7, 8^. Pericytes are also present in the normal lung^9^, and resident mesenchymal progenitors with a pericyte-like phenotype were recently identified as lung myofibroblast precursors in bleomycin-induced pulmonary fibrosis^10^. Interestingly, pericytes have recently been described as an important component of the tumor microenvironment^6, 11^. However, their contribution to an activated stroma and in non-small cell lung cancer (NSCLC) is currently not clear.

Because of their anatomical location on the abluminal side of capillaries and microvessels, pericytes carry out essential functions with respect to vessel guidance, basement membrane production, vascular tone and quiescence^12, 13^. High levels of proangiogenic and migratory growth factors present in the tumor microenvironment (TME) also affect the vascular compartment^14^. Indeed, abnormally organized, unstable and leaky tumor vessels, as a result of an aberrant basement membrane and poor pericyte coverage, are a hallmark feature of solid tumors^15, 16^ and play a prominent role in metastasis^17^. Recent evidence in a murine model of breast cancer showed that PDGFRβ+ pericytes differentially regulate tumor progression^18^. Pharmacological or genetic ablation of PDGFRβ+ vascular pericytes at early stages of cancer development reduced lung metastases, whereas increased metastases were observed when pericytes were depleted at later stages in tumor development. Blockage of angiopoietin-2 (Ang-2) together with pericyte depletion was able to reduce vascular leakage, vessel diameter and lung metastases. In a different study, pericytes were shown to regulate myeloid-derived suppressor cell (MDSC) recruitment. Experimentally induced tumors in pericyte-deficient mice correlated with increased numbers of locally recruited MDSCs^19^. In a cohort of human breast cancer specimen, reduced pericyte gene expression together with increased MDSC markers correlated with poor clinical outcome. Together, these findings point to an important role of pericytes in modulating solid tumor progression and host immunosurveillance.

Since pericytes occupy a perivascular location within the tumor microvasculature, they represent a potential stromal target for cancer therapy in solid tumors. Here, we investigated the presence and functional properties of local perivascular cells/pericytes in patients with early-stage resectable NSCLC. We prospectively isolated perivascular-like cells from human NSCLC specimens and characterized surface marker and cytokine expression. To investigate their functional role in supporting microvessels, perivascular-like cells were seeded in a microfluidic device, and vessel morphology and permeability were assessed.

## Results

### Identification and isolation of Lin-EpCAM-CD73+CD90+ cells in NSCLC

**Figure 1:**
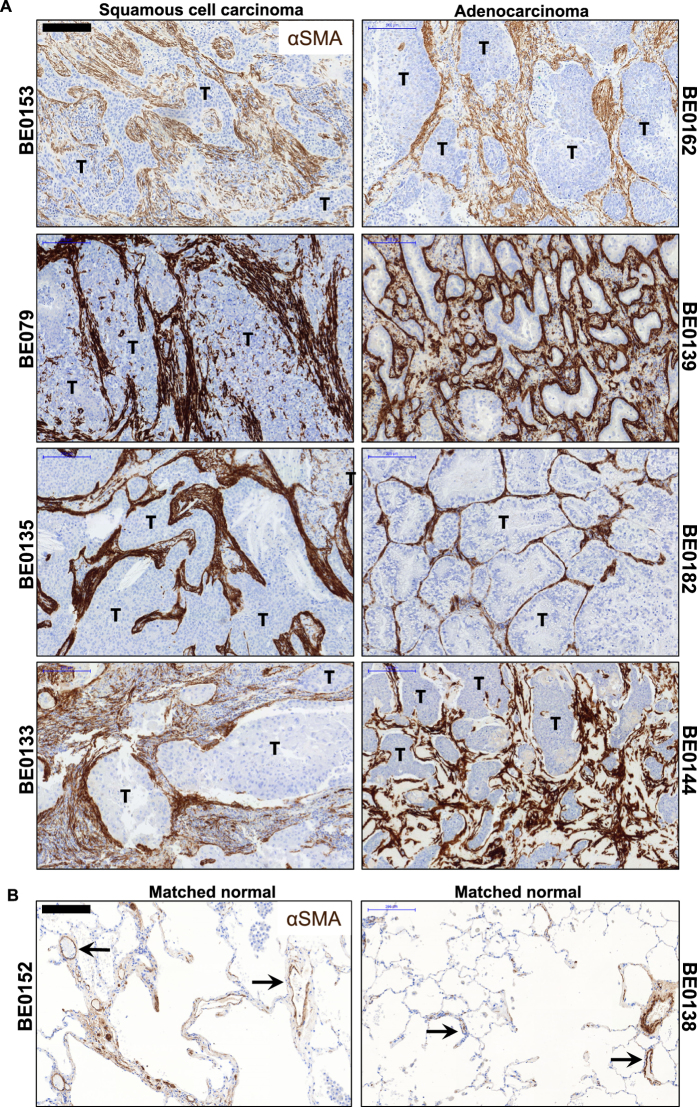
Evidence of activated stroma in NSCLC. (**A**) Representative immunohistochemistry sections from lung squamous cell carcinoma (left panels, n = 4) and adenocarcinoma (right panels, n = 4) patient specimens show active cancer-associated stroma throughout the tumor foci (T), featuring markedly increased αSMA positivity. Scale bar 200 µm. (**B**) Representative sections taken from nonadjacent normal section of the lung from matched tumor specimens. In the distal area of the uninvolved normal lung, aSMA+ areas can be seen primarily surrounding both small and large vessels (black arrows, n = 2). Scale bar 200 µm. See related supplemental Figure S1.

**Figure 2:**
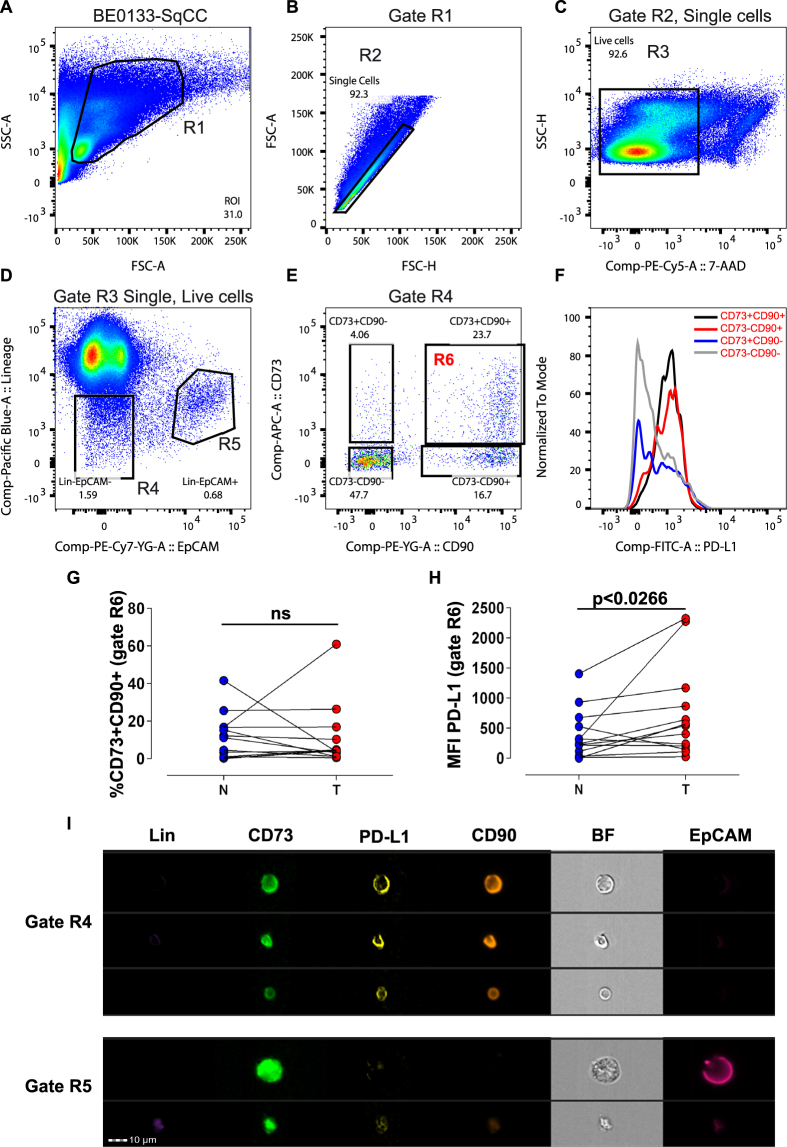
Prospective isolation of mesenchymal cell subset in NSCLC with increased expression of PD-L1. (**A**–**E**) Representative pseudocolor dot plots from a patient specimen (BE0133, squamous cell carcinoma) showing the gating strategy to identify clusters of mesenchymal cells within the NSCLC tumor specimen. Cells (R1 gate) initially displayed on a SSC/FSC color density plot (**A**) subgated to select single cells based on FSC-Area versus FSC-Height (R2 gate) (**B**), were further subgated for identification of live (7-AAD negative) cells (R3 gate) (**C**). Single, live cells (Gate R3) were displayed on a bivariate plot showing the presence of a cluster of Lin- cells that lacked the epithelial cell adhesion marker EpCAM (CD326) (R4 gate) and a cluster positive for EpCAM (R5 gate) (**D**). Lin-EpCAM- cells (Gate R4) displayed as a bivariate plot to identify CD73 and CD90 subset of cells (**E**). The expression of each subset for PD-L1 is shown on a histogram plot (**F**) (black: CD73+CD90+, red: CD73-CD90+, blue: CD73+CD90−, gray: CD73-CD90−). (**G**) Scatter plot showing the Lin-EpCAM-CD73+CD90+ cells (gate R6), as a percentage (%) of total counted events determined from Gate R4 (n = 13, biological replicates). (**H**) Scatter plot showing the mean fluorescence intensity (MFI) for PD-L1 in the Lin-EpCAM-CD73+CD90+ mesenchymal cell subset (gate R6) in tumor (T) versus matched nonadjacent uninvolved tissue (N) (n = 13, biological replicates). (**I**) Representative images obtained from a single lung adenocarcinoma cell using ImageStream®. Statistical analysis in G and H by Student t-test, two-tailed, for comparison of paired parametric data. All tests were two-tailed. *p < 0.05 were considered significant. See related supplementary data Figures S2–4.

We previously demonstrated that mesenchymal cells with a pericyte-like phenotype in the normal human lung support microvessel formation using an *in vitro* microfabricated platform^20^. Since pericytes are a key compartment of the tumor stroma and their coverage and function is often deficient in the tumor microvasculature, we were interested in determining their presence and functional state in early-stage, resectable NSCLC. First, to evaluate the amount and location of activated stroma *in situ*, NSCLC specimens from both histological subtypes (Table S1) were stained with αSMA. We could detect αSMA+ cells present in the tumor foci with widespread distribution and intensity. Moreover, aSMA+ layers can be seen often surrounding tumor clusters (Fig. 1A and Figure S1A). In the matched nonadjacent uninvolved lung tissue, aSMA+ cells also were detected; however, the majority were localized primarily to small and large vessels in the distal airway (Fig. 1B and Figure S1B). CAFs are thought to be the main source of αSMA+ cells in solid tumors. To gain a better understanding of the mesenchymal cell that my serve as a source of myofibroblast precursor cells giving rise to activated stroma in NSCLC, we designed a multicolor antibody panel for multiparameter fluorescently activated cell sorting (FACS) to separate mesenchymal from epithelial and hematopoietic/endothelial cells. Using this panel, we were able to identify and isolate a mesenchymal-like cell subset from both lung adenocarcinoma and squamous cell carcinoma patient samples (Table S1) along with matched normal lung tissue (Fig. 2). Within the mesenchymal compartment of both histological subtypes, represented as Lin-EpCAM- gate R4 (Fig. 2D; see Figure S2A–D for full gating strategy using FMO to determine boundaries), we identified three main population clusters based on differential expression of CD73 (ecto-5′-nucleotidase) and the membrane glycoprotein CD90 (Thy-1) (Fig. 2E), two markers co-expressed by mesenchymal stromal/stem cells (MSCs), as well as perivascular and nonperivascular cells. In addition, each subtype was further subgated onto a histogram plot showing expression of the inhibitory programmed death 1 (PD-1) receptor, PD-L1 (Fig. 2F). The predominant mesenchymal cluster was double positive for CD73 and CD90 (11.52 ± 12.13, normal tissue N; 10.70 ± 16.53, tumor-affected tissue T, n = 13, ns; Fig. 2G). These populations were sorted. Despite no difference in the presence of the Lin-EpCAM-CD73+CD90+ mesenchymal subset between tumor and matched normal, there was a significant difference in the expression level of PD-L1 (MFI 724 ± 767 versus 384 ± 409, respectively, n = 13, *p* < 0.0266, Fig. 2H). A backgating analysis confirmed the positon of the R6 gate used to prospectively isolate the cells of interest in gate R6 (Figure S3). In tissue from a single lung adenocarcinoma patient we used ImageStream® analysis to provide high-resolution imagery with fluorescent sensitivity. Based on bright field imaging (BF), the small cell size is compatible with a perivascular-like cell and demonstrates co-expression of CD73, and CD90 with PD-L1, while lacking lineage markers (CD45, CD14, CD31) and EpCAM, whereas EpCAM+ cells found in gate R5 are larger (Fig. 2I and see also Figure S4A,B). Taken together, these results show the existence of a subset of mesenchymal cells of small size that overexpress PD-L1 within the tumor microenvironment in NSCLC.

### Tumor-derived Lin-EpCAM-CD73+CD90+ cells express common perivascular and nonperivascular markers and display an altered mesenchymal differentiation potential

**Figure 3:**
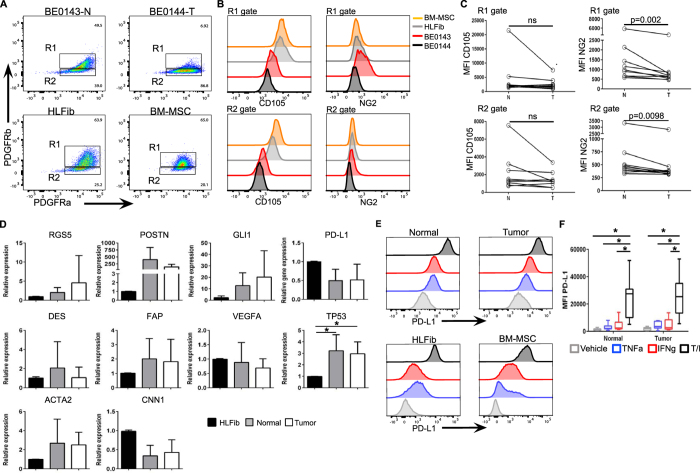
Isolated Lin-EpCAM-CD73+CD90+ mesenchymal cell subset display a perivascular-like phenotype. (**A**) Representative bivariate flow cytometric plots showing the expression of PDGFR-beta PDGFR-alpha in Lin-EpCAM-CD73+CD90+ cells isolated from tumor and matched normal (upper panels) specimens, as well as human lung fibroblasts (HLFib, CCD-16Lu, ATCC® CCL-204™) and BM-MSC (lower panels). (**B**) Expression of CD105 (left panels) and NG2 (right panels) for the subsets of cells in gate R1 and R2 demonstrated in histogram overlay. (**C**) Scatter plots showing the MFI of CD105 and NG2 in the cell subsets R1 and R2 (n = 10, biological replicates, see Figure S5 for FMO controls for setting cell boundaries). (**D**) mRNA expression of selected genes of various mesenchymal markers and functional categories specific to the lung in sorted Lin-EpCAM-CD73+CD90+ (n = 8, biological replicates). HLFib was set at one. (**E**) Representative histogram overlay showing the change in expression of PD-L1 in normal and tumor-derived Lin-EpCAM-CD73+CD90+ cells (upper panels) in response to exposure to proinflammatory cytokines, as well as in HLFib and BM-MSC (lower panels). (**F**) Bar graphs showing the change in MFI for PD-L1 (n = 8, biological replicates). Data in (**D** and **F**) are presented as mean ± SD. Error bars show SD. Statistical analysis in C by Student t-test, two-tailed, for comparison of paired or unpaired parametric data. All tests were two-tailed. Statistical analysis of means for more than two groups in (**D** and **F**) were by one-way ANOVA and multiple comparisons using post hoc Newman-Keuls test. *p < 0.05 were considered significant. See related supplementary data Figures S5–S7.

Lin-EpCAM-CD73+CD90+ mesenchymal cells prospectively isolated using FACS were amenable to expansion in culture, and possess a perivascular-like morphology consisting of a central nucleus with branching and small cell size (see Figure S5A). Following expansion, flow cytometric analysis revealed the presence of PDGFRα (CD140a) and PDGFRβ (CD140b) on tumor-derived Lin-EpCAM-CD73+CD90+ mesenchymal cells and their normal counterparts (Fig. 3A, top panels, see also Figure S5B–H), as well as human lung fibroblasts (HLFib) and bone marrow-derived MSCs (BM-MSC) (Fig. 3A, bottom panels). Further analysis of these two subsets of cells (R1 and R2) confirmed the presence of another common mesenchymal marker CD105 (Endoglin), as well as the pericyte marker neural glial antigen (NG2) (Fig. 3B). There was a trend towards a decrease in CD105 in the tumor-derived population. However, this did not reach statistical significance (Fig. 3C, left panels). NG2 expression was decreased on tumor-derived PDGFRb+PDFGRa+ subset (R1 gate) compared to their matched normal counterpart (1029 ± 1229 versus 1504 ± 1598, respectively, n = 10 biological replicates; Fig. 3C, upper right panel). The same trend was found for the PDGFRb-PDGFRa+ subset (R2 gate, 607 ± 779 versus 740 ± 915; Fig. 3C, lower right panel). The expression levels for CXCR4, ICAM, GD2 and CD146 between tumor versus matched normal was heterogeneous and did not differ (data not shown). In two donors, we confirm the lack of expression of epithelial specific markers EpCAM and E-Cadherin on Lin-EpCAM-CD73+CD90+ cells (see Figure S6).

At the molecular level, FACS-sorted Lin-EpCAM-CD73+CD90+ cells expressed common pericyte and nonperivascular markers RGS5, desmin, Gli1, αSMA and calponin (Fig. 3D). We found an upregulation of periostin (POSTN) and tumor suppressor protein p53 (TP53) in the cells isolated from NSCLC tissue. Based on our finding that tumor derived Lin-EpCAM-CD73+CD90+ cells express elevated levels of PD-L1, we also examined whether PD-L1 can be further regulated following exposure to pro-inflammatory cytokines. We found that both tumor-derived Lin-EpCAM-CD73+CD90+ cells and their matched normal counterparts upregulate PD-L1 following exposure to TNFα and IFNγ, as shown in histograms overlays (Fig. 3E, top panels). For comparison, we also exposed normal human HLFib and BM-MSCs to TNFα and IFNγ and measure the change in PD-L1, also shown as histogram overlays (Fig. 3E, bottom panels). Although TNFα and IFNγ both increased PD-L1 expression (4202 ± 2500 and 4724 ± 4155, respectively, Fig. 3E and F), it was only the combination TNFα/IFNγ (25818 ± 14131, p < 0.0001) that resulted in a significant increase in PD-L1 expression in tumor-derived Lin-EpCAM-CD73+CD90+ cells. However, there was no difference compared to their matched normal counterpart (Fig. 3F).

Since perivascular cells in general possess mesenchymal-like properties, we next wanted to assess the differentiation potential of tumor-derived Lin-EpCAM-CD73+CD90+ cells. When grown in adipogenic induction media, pericyte-like cells from the normal tissue were able to generate lipid droplets that stained positive for Oil Red O (upper panel, Figure S7A). The adipogenic potential of tumor-derived Lin-EpCAM-CD73+CD90+ cells was not different from cells obtained from the nonadjacent normal; however, adipogenesis was diminished compared to BM-MSCs from a healthy donor (lower panel, Figure S7A). This was not the case when tumor-derived cells were grown in an osteogenic induction media. The uptake of Alizarin Red S, a marker used to indicate calcium-rich mineralization of the cell matrix, was more robust in the tumor-derived population (right panel, Figure S7B) compared to their matched normal counterpart (left panel, Figure S7B). As a positive control, we show the generation of lipid droplets and Alizarin Red S stain for mesenchymal stromal cells derived from the bone marrow of a healthy donor (Figure S7C).

### Induction of αSMA in tumor-derived Lin-EpCAM-CD73+CD90+ mesenchymal cells in response to TGF-β1 but not Jagged1

**Figure 4:**
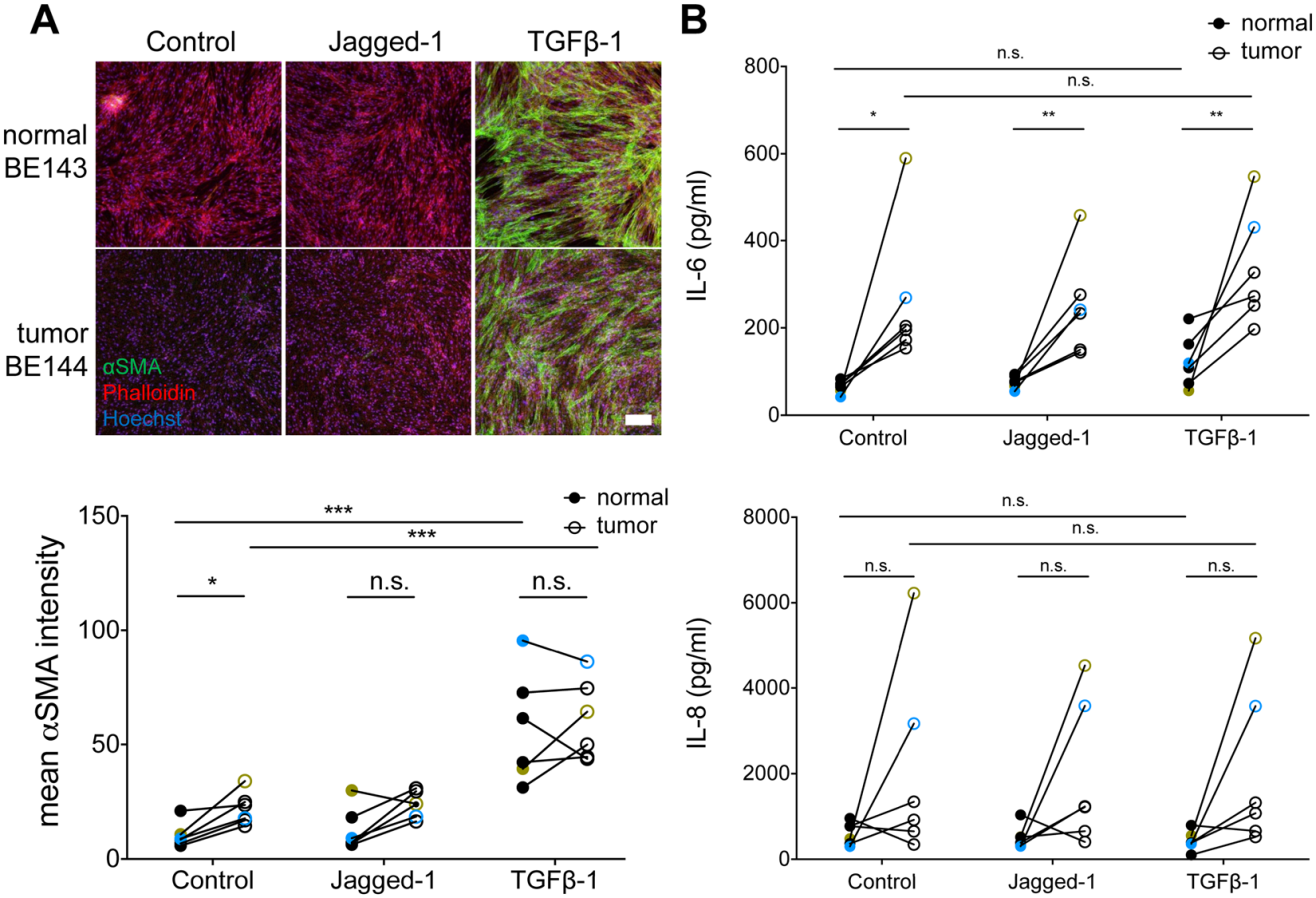
Upregulation of αSMA and cytokine release in response to TGF-β1. (**A**) Images of normal and tumor-derived Lin-EpCAM-CD73+CD90+ cells stained for αSMA, phalloidin and Hoechst after 3 days of treatment with 10 ng/ml TGF-β1, 50 ng/ml Jagged1 or serum-free conditions (scale bar: 200 μm) and quantification of mean αSMA signal intensity. (**B**) Scatter plots showing levels of IL-6 and IL-8 measured by ELISA in six matched samples of Lin-EpCAM-CD73+CD90+ cells after one day of exposure to TGF-β1, Jagged1 or control treatment. Measurements from patient-samples BE132-133 are marked in yellow and BE143-144 in blue, to highlight the high levels of both IL-6 and IL-8 secretion. n = 6 matched samples, three replicates were measured for each sample. Statistical analysis in A and B by Student t-test for comparison of paired parametric data. All tests were two-tailed. *p < 0.05 were considered significant. See related supplementary data Figure S8.

TGF-β1 is a well-known promoter of pericyte and smooth muscle cell proliferation and differentiation^21^ and is also one of the main triggers for the conversion of fibroblasts to CAFs known to be present in solid tumors^22^. In contrast, Notch signaling *via* Jagged1 is required for differentiation into mature pericytes^23, 24^. Exposure of tumor-derived Lin-EpCAM-CD73+CD90+ cells over a three-day period to TGF-β1, but not Jagged1, induced the expression of αSMA (n = 6 matched samples, p < 0.001) (Fig. 4A). Interestingly, there was inter-patient variability regarding induction of αSMA expression in untreated samples (Figure S8). Furthermore, we found a small elevation of αSMA expression in untreated tumor-derived perivascular-like cells compared to normal counterparts (n = 6, p = 0.01, Fig. 4A).

### Tumor-derived Lin-EpCAM-CD73+CD90+ mesenchymal cells show elevated basal secretion of IL-6

Next, we assessed the effect of activating tumor-derived pericytes with TGF-β1 on immunogenic and angiogenic cytokine secretion. Interestingly, we found that perivascular-like cells from tumor tissue secreted high levels of IL-6 compared to the matched normal cells. This was true under basal conditions (n = 6, p = 0.014), when stimulated with Jagged1 (n = 6, p = 0.004) or TGF-β1 (n = 6, p = 0.004, Fig. 4B). There was a trend towards IL-6 upregulation upon exposure to TGF-β1. It was smaller in tumor-derived perivascular-like cells (p = 0.094) compared to their normal counterpart (p = 0.063). The two tumor-derived samples having high IL-6 levels also showed increased IL-8 secretion (BE133 marked in yellow, and BE144 marked in blue), but this effect was not observed in all matched samples (p = 0.136 for control, p = 0.070 for Jagged-1, p = 0.063 for TGF- β1, n = 6).

### Tumor-derived Lin-EpCAM-CD73+CD90+ mesenchymal cells promote formation of vessels with enhanced vascular permeability

**Figure 5:**
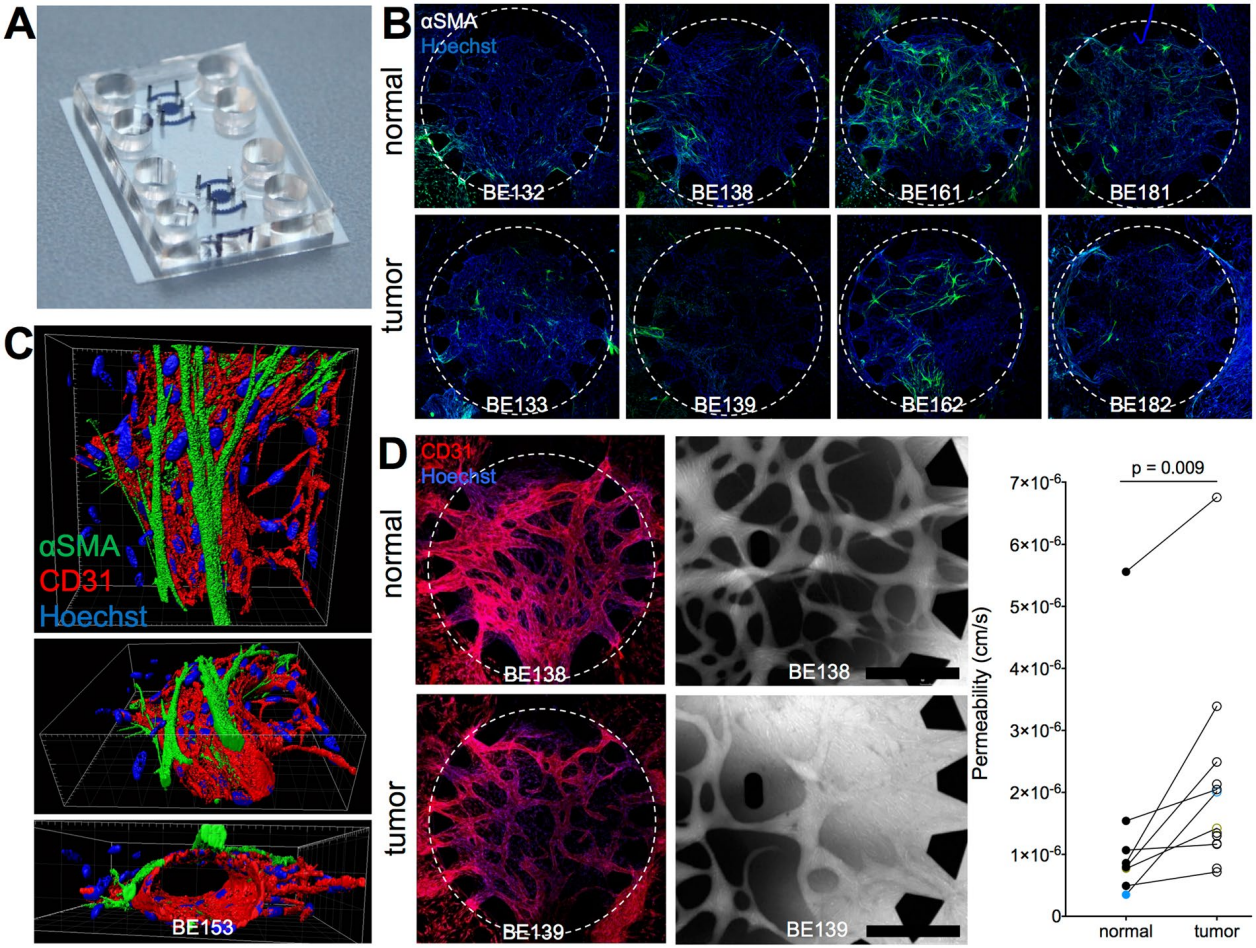
Microvessel formation, permeability and αSMA expression in surrounding pericytes. (**A**) A microfluidic chip with two round chambers for microvessel formation flanked by side channels for pericyte seeding (all chambers with cells and gel are marked in blue). (**B**) Representative images of a matched sample show αSMA + pericytes (green) in the microvascular chamber after one week in culture, counterstained with Hoechst (blue). Microvascular chambers are marked with a dotted line, diameter 2.4 mm. (**C**) 3D rendering of a αSMA + pericyte located on the abluminal surface of a CD31-stained microvessel. Scale bar: 30 μm. (**D**) Left: Endothelial microvascular networks continuously expressing CD31 (red) throughout the central chamber with open entrances between pillars. These networks form both when supported with normal (top) or tumor-derived (bottom) Lin-EpCAM-CD73+CD90+ cells. Center: Fluorescent 70 kDa RITC dextran (greyscale) show perfusability of microvascular networks and higher leakage of tumor PC-supported vessels compared to normal PC-vessels after 10 minutes. Scale bars: 500 μm. Right: Vascular permeability was measured with normal (n = 8) and tumor (n = 13) Lin-EpCAM-CD73+CD90+ cells (1 to 4 technical replicates per sample). Measurements from BE132-133 are marked in yellow and BE143-144 in blue. Statistical analysis in D by Student t-test, two-tailed, for comparison of paired parametric data. All tests were two-tailed. *p < 0.05 were considered significant. See related supplementary data Figure S9.

Investigating the functional role of perivascular cells necessitates a microenvironment that reproduces the essential steps involved in blood vessel formation, such as pericyte recruitment and vessel stabilization. We previously developed such an environment using a microfluidic chip, where endothelial cells (EC) and lung pericyte-like cells (PC) suspended in a fibrin matrix self-assembled to microvessels in a central chamber (Fig. 5A). Side chambers filled with Lin-EpCAM-CD73+CD90+ cells only guided the vessel formation such that open, perfusable networks formed^20^. Here, endothelial cells self-assembled forming microvascular networks within 7 days when co-cultured with tumor-derived pericytes or their matched normal counterparts in a fibrin matrix inside the microfluidic chip. The endothelial cells built a continuous and stable vascular network as confirmed by PECAM-1 staining (Fig. 5B, top). The presence of Lin-EpCAM-CD73+CD90+ cells derived from the tumor or their normal counterparts was necessary and sufficient to stabilize the endothelial microvascular network. The microvessels were accessible from the flow channels when mesenchymal cells were seeded in the side chambers. We previously reported that perfusability of microvessels is dependent on the presence of pericytes in the side chambers^20^. Therefore, tumor-derived Lin-EpCAM-CD73+CD90+ cells retain the capacity to guide microvessel patterning and led to perfusable microvessels in a similar fashion as their normal counterparts.

Next, we wanted to determine if tumor-derived Lin-EpCAM-CD73+CD90+ cells when in contact with endothelial cells adopt a mural cell fate with respect to location, differentiation and function. We investigated αSMA expression in the microfluidic chip. Fig. 5B center shows αSMA positive pericytes (green) in close proximity to microvessels (red). Three-dimensional rendering of the vascular network (Fig. 5C) shows the abluminal location of pericytes wrapping around endothelial microvessels (see supplemental Figure S5 and see supplemental movie S1). Comparing αSMA expression in vessels supported by normal versus tumor-derived Lin-EpCAM-CD73+CD90+ cells respectively, we noted that the number of perivascular-like cells expressing αSMA was variable between patients with no consistent difference between tumor and matched normal samples (Fig. 5B and Figure S9).

To compare the effects of Lin-EpCAM-CD73+CD90+ cells on microvascular stability, following perfusion of the 3D vessel structures with fluorescently labeled 70 kDa dextran, the signal intensity in the extravascular space over time was measured. The leakage of fluorescent dye into the extravascular space is a measure of vessel permeability, as shown in the representative examples in Fig. 5D (upper panels, see Supplemental movies S2 and S3). We found that microvessels in contact with tumor-derived Lin-EpCAM-CD73+CD90+ cells were more permeable compared to vessels lined with normal (p = 0.0091, normal PC: n = 8, tumor PC: n = 13, Fig. 5D, lower left panel). These findings demonstrate that the predominant mesenchymal population in the lung tumor microenvironment most likely identifies a pool of cells with perivascular-like properties.

## Discussion

Here, we show the presence of mesenchymal cells with the immunophenotypic profile Lin-EpCAM-CD73+CD90+ in early stage resectable NSCLC were associated with a higher baseline expression of the immunosuppressive ligand PD-L1, as well as an enhanced basal IL-6 secretion compared to their normal counterpart derived from matched uninvolved lung. Furthermore, using an organotypic model of perfusable microvessels, we demonstrate that tumor-derived Lin-EpCAM-CD73+CD90+ mesenchymal cells (herein referred to as perivascular-like) specimens support microvessel formation, upregulate αSMA upon contact with microvessels, but lead to higher microvascular permeability compared to their matched normal counterparts.

Perivascular cells (pericytes or mural cells) are essential for the stabilization of blood vessels in solid organs^12^. However, deficient coverage in the tumor vasculature contributes to tumor progression and metastasis, which may be circumvented by targeting tumor perivascular cells and normalizing the tumor vasculature^25^. The *in vitro* microvascular model used in our study comprises key features of vessel formation and stabilization allowing us to perform permeability measurements. We found that both normal and tumor-derived perivascular-like cells were able to guide vessel formation indicating an intact cross-talk required during the initial steps of new blood vessel formation. Both normal and tumor-derived perivascular-like cells were found to upregulate αSMA, while assuming an abluminal position in close proximity to patent microvessels, consistent with a pericyte function.

Despite this, vessels lined with tumor perivascular-like cells were leaky, which is in agreement with animal and human data^26^. Deficient perivascular coverage of tumor blood vessels in a PDGFRβ−/− mouse model of breast cancer resulted in recruitment of MDSCs contributing to an immunosuppressive tumor microenvironment mediated by the induction of the protumorigenic cytokine IL-6 in malignant cells^19^. Recent evidence in NSCLC cell lines with EGFR-activating mutations showed that blockade of IL-6 signaling with metformin was able to overcome chemoresistance to tyrosine kinase inhibitors^27^. Besides its direct protumorigenic effects on tumor cells via STAT3/Akt pathways, IL-6 signaling has indirect effects by promoting an immunosuppressive tumor microenvironment, which has been described in a Kras-induced lung cancer mouse model^28^. To date, the protumorigenic source of IL-6 in both preclinical animal models of lung cancer and human samples has been shown to be epithelial-derived^29^.

There is a clear association between lung inflammation and the tumorigenic effects of IL-6^30^. However, the source of altered cytokine secretion is studied from the standpoint of the tumor cells. We show for the first time a perivascular source of IL-6 in human NSCLC. Besides exhibiting an elevated basal level of IL-6, tumor perivascular-like cells also show an enhanced expression of the IFNγ-inducible immunosuppressive ligand PD-L1, which in advanced NSCLC may represent a novel biomarker for immune checkpoint blockade therapy^31, 32^. To our knowledge, this is the first report of enhanced IL-6 together with PD-L1 expression in a mesenchymal cell population in early stage resectable NSCLC. IL-6 has recently been shown to play a role in pathogenic angiogenesis by regulating, in part, the expression of Ang-2^33^. In conjunction with pericytes, Ang-2 also was shown to contribute to tumor progression and metastasis. Depletion of pericytes in hypoxic tumors led to increased metastases, whereas concomitant blocking of Ang-2 normalized the vessels and decreased metastases^18^. Although not investigated here, it is possible that the elevated IL-6 levels we observed in tumor pericytes leads to vessel activation and pericyte detachment via Ang-2 in the present microvascular model. We also found elevated basal levels of CXCL8/IL-8, a chemoattractant for neutrophils, produced by macrophages^34^ and promoter of endothelial proliferation and MMP secretion^35^. However, there was significant patient heterogeneity. The role of CXCL8/IL-8 in this setting is not clear.

In this study, we compared tumor-derived perivascular-like cells to their matched normal counterpart. Although the normal cells are recovered from a section of the lung distant from the tumor foci, it is impossible to rule out that the ongoing inflammation and stress associated with malignancy does not poses any long lasting effect on these cells as well. Moreover, our study does not address the ontogeny of the tumor perivascular-like cells. Whether perivascular cells in solid tumors are recruited from a replicating source of locally derived cells or an extrapulmonary source such as bone marrow MSCs is still under debate^36, 37^. Previous fate-mapping experiments show that replicating local fibroblasts as the major source of myofibroblasts contributing to fibrosis^38^. In the lung, a heterogeneous population of stromal cells of mesenchymal origin but not epithelial cells provides a source of myofibroblasts following bleomycin injury^39^. Schreiber and colleagues demonstrate that tumor-associated fibroblasts do not originate from circulating cells but from local a source^40^. The authors speculate that resident mesenchymal cells such as MSCs or pericytes could provide a pool of cells that transition to fibroblasts. In a separate study, Cao and colleagues show that in solid tumors NG2+ vascular pericytes detach from tumor vessels and transition to fibroblasts leading to enhanced tumor growth and metastasis^41^. Although both studies were based on fate-mapping studies mice, they provide additional evidence that perivascular cells may represent a unique target in the treatment of solid tumors. Notwithstanding, one of the main hurdles in the field of human pericyte biology is no one single marker can be used to identify pericytes, as most markers are promiscuous and also present on other mesenchymal cell types, such as MSCs or stromal fibroblasts^42^. To address this, we combined a mixture of criteria^12^ such as cell size, morphology, surface marker expression and *in vitro* functional tests to retrospectively identify and characterize perivascular-like cells in early-stage, resectable NSCLC. Further, their ability to expand and undergo adipogenic and osteogenic differentiation, as well as differentiate into αSMA+ myofibroblasts in response to TGF-β1 suggests that this cell source may likely serve as a viable pool of myofibroblast precursor cells in the tumor microenvironment.

The microfluidic platform for vascular assembly allows studying vessel formation and maturation with endothelial-perivascular cell interactions *in vitro*. Despite its ease of use and functional readouts such as vascular permeability, the system holds some limitations. For example, using fibrin as extracellular matrix mimics an activating wound-healing environment and does not contain ECM components from the lung. However, with time, cells secrete basement membrane components such as collagen IV and laminin. Also, the experimenter decides on the types of cells used in the model (here, endothelial cells and mesenchymal cells). On the one hand this can be a disadvantage, as not all possible interactions found *in vivo* may be reproduced. On the other hand it holds the advantage that only functions specific to a cell type in relation to another may be studied, disregarding all other factors.

In summary, our work demonstrates the presence of perivascular-like cells within the tumor microenvironment in early-stage, resectable NSCLC. Furthermore, we show that perivascular-like cells retain functional abnormalities in cytokine secretion and support of 3D microvessels in a microfluidic platform. This data suggests that tumor perivascular-like cells not only inadequately support vascular structures, but may also actively contribute to an immunosuppressive tumor microenvironment in a paracrine fashion. Intriguingly, the perivascular-like cells retain certain abnormalities despite being removed from the tumor microenvironment and expanded *in vitro*. The prevailing view is that the mesenchymal or stromal compartment of solid tumors remains genetically stable. However, it was recently reported that stromal cells may acquire defects such as concomitant loss of p53, which could affect their function^43^. In our study, we found p53 to be upregulated in tumor-derived Lin-EpCAM-CD73+CD90+ cells, as well as nonadjacent normal, compared to normal human lung fibroblasts. Li and colleagues^44^ demonstrated that epigenetic silencing of miRNAs in CAFs promotes their production of IL-6/PGE2 promoting tumor growth. However, whether miRNAs might also be involved in our observations require further investigation. Interestingly, unlike changes in IL-6 and vascular defects *in vitro*, the enhanced PD-L1 expression in perivascular-like cells detected *in vivo* was not maintained when cells were placed in culture. The reasons for this are presently not known. These findings underscore the need for additional studies aimed at uncovering which factors within the tumor microenvironment (i.e. hypoxia, inflammatory and oxidative stress, DNA damage) shape the biology of perivascular-like cells and how this in turn may enhance an already immunosuppressive tumor microenvironment in solid tumors of the lung in humans.

### Significance

There is mounting evidence supporting a central role of pericytes in tumor progression and metastasis. Here, using a mixture of criteria we show for the first time that perivascular-like cells in NSCLC retain functional abnormalities in cytokine secretion and support of microvessels *in vitro*. This data suggests that tumor perivascular cells not only inadequately support vascular structures, but also may actively modulate the inflammatory and immune response and represent a novel stromal cancer target in the setting of NSCLC.

## Methods

### Clinical sample collection and processing

Lung tumors samples were obtained from patients following surgical resection for NSCLC at the Inselspital, Bern university Hospital (n = 13, Table S1). All patients gave informed written consent for usage of surgical material for research purposes, which was approved by Ethics Commission of the Canton of Bern (KEK-BE:042/2015). All procedures were carried out in accordance with institutional guidelines from the Canton of Bern. The specimens were dissected by a pathologist (SB) and samples from the tumor center as well as matched non-tumorous tissue collected for further investigation. Following dissociation of the lung tissue to single cell suspension in a collagenase solution (0.1% collagenase I and 0.25% collagenase II) (Worthington Biochemical, Lakewood, NJ, USA) in 2% FBS (Invitrogen, Carlsbad, CA, USA). Tissue digestion was halted by addition of 10% FBS (Invitrogen). To generate single cells, the digested tissue sample was filtered sequentially through a 100 µm followed by 40 µm cell strainer (BD Falcon). Red blood cells were lysed by incubating single cell suspension in RBC lysis buffer (BioLegend, San Diego, CA, USA) for 10 minutes at room temperature. Following lysis, cells were resuspended in PBS and centrifuged at 600 g for 15 minutes. Cells were processed for fluorescence-activated cell sorting (FACS) and analysis, as described below. Routinely processed formalin fixed and paraffin embedded tissue of a subset of patients was cut at 3 μm and immunohistochemically stained on the automated system BOND RX® (Leica Biosystems, Newcastle, UK) using an anti-human smooth muscle alpha actin (αSMA) mouse monoclonal antibody (clone 1A4, 1:8000 dilution, Sigma Aldrich, Saint Louis, Missouri, USA) for 30 minutes at room temperature. Subsequently, the secondary antibody using Bond Polymer Refine Kit (Leica Biosystems) was applied for 15 minutes and the slides incubated with the chromogen DAB (3-3′-Diaminobenzidine) for 8 minutes.

### Fluorescence-activated cell sorting, analysis and primary cell culture

To identify and prospectively isolate mesenchymal and epithelial cell subsets in the developing lung, single cells were resuspended in staining buffer (2% FBS/1 mM EDTA/0.09% sodium azide). Following Fc block (eBioscience, San Diego, CA, USA), incubated with a panel of fluorescently conjugated human monoclonal antibodies directed at the following epitopes: CD45, CD14, CD31, CD235a, CD73, CD90, PD-L1 and EpCAM (see Table S2 for the respective conjugated fluorochrome). To exclude dead cells, 7-AAD was added prior to sorting. Using a BD FACS Aria III or BD FACS Aria, cells were sorted directly into collection buffer containing 20% FBS. Proper placement of gates was determined using fluorescence minus one strategy^45, 46^. Prospectively isolated mesenchymal cell subsets were plated on regular tissue culture plates coated with 0.2% gelation solution (Sigma Aldrich). Cells were expanded in chemically defined growth medium consisting of αMEM with ribonucleosides (Sigma Aldrich) supplemented with 1% FBS (Invitrogen), 200 mM L-glutamine (Gibco/Invitrogen), 10 ng/mL of recombinant human fibroblast growth factor 2 (FGF2, Invitrogen), 20 ng/ml of recombinant human epidermal growth factor (EGF, Invitrogen), human insulin (1.25 mg, Sigma Aldrich) and 1% antibiotics (Invitrogen). Cells were maintained in a humidified 37 °C low oxygen (3%O2) incubator in 5%CO2. On day 6 after plating, the medium was carefully aspirated and replaced with fresh medium and regular media changes were performed biweekly. Cells were maintained in a humidified 37 °C low oxygen (3%O_2_) incubator. Once cells reached confluence, they were harvested using 1x solution of TrypLE (Invitrogen) and reseeded for expansion and a portion of the cells were lysed with RLT buffer (Qiagen, Hilden, Germany) and stored at −80 °C for RNA isolation at a later time point. In a separate experiment, single cells from single lung adenocarcinoma specimen were processed for ImageStream® analysis. Briefly, single cells were stained with the following antibodies EpCAM-PE-Cy7, CD73-FITC, CD90-PE-Texas Red, PD-L1-PE and dump channel for lineage markers with live/dead fixable dye (eFluor450). Following staining, cells were imaged using Amnis® imaging flow cytometer (EMD Millipore, Billerica, MA, USA) to characterize cell morphology at a high resolution providing information regarding bright field, side scatter and co-localization of markers. To quantify the level of PD-L1 expression, fcs files were imported into FlowJo ver10.1 (TreeStar) for analyzing flow cytometry data. Briefly, following selection of single, live cells, cells of interest were displayed on a bivariate plot for CD73 and CD90. Expression for PD-L1 for the various populations were displayed as histogram overlays (see Fig. 2F). PD-L1 expression was quantified as geometric mean fluorescence intensity (MFI) in FlowJo from gate R6, which was the prospectively isolated population. For analysis, a minimum of 5 × 10^5^ to 1 × 10^6^ cells were collected.

### Immunophenotype using flow cytometry

Following expansion, FACS sorted mesenchymal cell subsets were harvested and re-suspended in FACS staining buffer. Following Fc block, cells were incubated with the following fluorescently conjugated human monoclonal antibodies used to detect mesenchymal lineages: PDGFRα-PE, PDGFRβ-APC, GD2-APC, NG2-FITC, CD105-PE-Cy7, CXCR4-BV711, CD146-FITC and CD54 (ICAM)-Pacific blue (see Table S2). For each antibody, a titration experiment was performed to determine the optimal dilution for the immunophenotypic analysis. Cells were incubated on ice in the dark for 30 minutes. To exclude dead cells and debris, 7-AAD was added. From two donors, we examined the expression of EpCAM-BV605™, E-Cadherin Alexa Fluor®488, CD90-PE Texas Red, CD61-APC on prospectively isolated cells. Cell acquisition was performed using a BD FACS LSRII. For analysis, a minimum 10,000 events were collected and analyzed using FlowJo software version ver10.1.

### Treatment with TGF-β1 and Jagged1

Tumor and matched normal Lin-EpCAM-CD73+CD90+ cells were harvested and seeded in glass slide chambers coated with 0.1% gelatin at 50,000 cells/well. 48 hours after seeding, cells were treated with 50 ng/ml recombinant human Jagged-1 (Peprotech, Rocky Hill, NJ, USA) and/or 10 ng/ml recombinant human TGF-β1 (Invitrogen) for three consecutive days. Media was saved and stored at −80 °C. Following treatment, cells were fixed with 4% paraformaldehyde (Sigma Aldrich) for 15 minutes. The permeabilization of cells was carried out with 0.1% Triton X-100 (Sigma Aldrich), followed by lock for 1 hour in 2% BSA (Sigma Aldrich) and stained with αSMA-FITC (Sigma Aldrich), Phalloidin-RITC (Molecular Probes, Eugene, OR, USA) and Hoechst 33342 (Invitrogen) for 2 hours. Samples were imaged using a Zeiss laser scanning microscope 710.

### ELISA

Media from the treatment with Jagged-1 and TGF-β1 was centrifuged at 500 g for 5 minutes, the supernatants recovered and examined for the release of IL-6 and IL-8 using ELISA (R&D Systems, Minneapolis, MN, USA), as described by the manufacturer.

### Differentiation assay

For adipogenic induction, 1 × 10^5^ Lin-EpCAM-CD73+CD90+ cells from the tumor and matched normal lung were plated per well in a 6-well dish in regular culture medium, as described above, and placed in a humidified chamber with 5%CO2 at 37 °C. After 24 hours, the wells were washed with PBS and fresh adipogenic induction medium (Invitrogen) was added. Medium was changed every 3 days and after 21 days cells were fixed with 4% paraformaldehyde and stained with Oil Red O (Sigma Aldrich) to detect formation of lipid droplets. In separate wells, RLT lysis buffer (Qiagen) was added and stored at −80 °C for generation of RNA at a later time point. For osteogenic differentiation, cells were plated at a low density in regular medium and after 24 hours were changed to osteogenic induction media (Invitrogen). Every 3 days, half-media changes were made. After 21 days, cells were fixed in 4% paraformaldehyde solution for 30 minutes and stained with 40 mM Alizarin Red S solution.

### Vasculogenesis on microfluidic chip

Human umbilical vein endothelial cells (Invitrogen) were cultured in EGM2 (Lonza, Walkersville, MD, USA) and used for experiments between passages four and six. Lin-EpCAM-CD73+CD90+ cells from patients were used up to passage four. Endothelial cells and mesenchymal cells were seeded in a fibrin scaffold into predefined compartments of a microfluidic chip, as previously described^20^. Briefly, both cell types were taken up and resuspended in a 2 U/ml thrombin (Biopur, Reinach, Switzerland) solution. The cell suspensions were first mixed together and then mixed 1:1 with fibrinogen (Sigma Aldrich) to a final concentration of 2.5 mg/ml fibrinogen, 5*10^6^ EC/ml and 2.5*10^6^ PC/ml in 1 U/ml thrombin. The mixture was immediately pipetted into the central chamber. For the side chambers, fibrinogen and the pericyte suspension were mixed to a final concentration of 2.5 mg/ml fibrinogen and 5*10^6^ PC/ml in 1 U/ml thrombin. After five minutes incubation at 37 °C for crosslinking, the flow channels were filled with 200 μl EGM2 per chip and a pressure drop of 1.2 mm H_2_O across the central chamber was established. The microfluidic chips were incubated in humidified petri dishes at 37 °C and 5%CO2 and medium was changed every second day.

### On-chip Permeability Measurement and Quantification

Permeability of the microvessels was assessed on day seven as previously described^20^. In short, the medium was removed from all reservoirs, and 100 μl of 1 mg/ml RITC-labeled 70 kDa dextran (Sigma Aldrich) was added to one reservoir and flowed through the vascular network. Fluorescence and bright field images were taken every 10 seconds for up to 10 minutes on a DMI 4000 fluorescence microscope. The microvessel permeability was calculated from the signal increase across vascular segments over time using with the following equation:

1 $$P=\frac{1}{{\rm{\Delta }}I}\cdot \frac{dI}{dt}\cdot \frac{r}{2}$$

where ΔI is the initial intensity increase, (dI/dt) the change of intensity over time and r the radius of a cylindrical segment. Five to seven segments were evaluated per chip; and two to six chips were evaluated for each patient. To compare populations, the Wilcoxon test was applied to matched samples and the Mann-Whitney test was applied in the rest of the cases.

### On-chip Immunostaining and Imaging

On day seven, EGM2 in the microfluidic chips was replaced with 200 μl PBS (Invitrogen) prior to fixation with 4% paraformaldehyde for 15 minutes. After three washings with PBS, the cells were permeabilized with 0.1% Triton X-100 (Sigma Aldrich) and blocked for one hour in 2% BSA (Sigma Aldrich) in PBS. Subsequently, the cells were either directly labeled with αSMA-FITC Clone 1A4 (Sigma Aldrich), Phalloidin-RITC (Molecular Probes, Eugene, Oregon, USA) and Hoechst 33342 (Invitrogen) in PBS, or incubated overnight with goat PECAM-1 (Santa Cruz Biotechnologies, San Diego, CA, USA), and after washing incubated with donkey ant-goat Alexa 546 secondary antibodies 1:500 (Molecular Probes) and directly labeled antibodies. The microfluidic chips were imaged on a Zeiss confocal laser scanning microscope 710. For signal quantification, the mean intensity on an area of 1.5 mm * 1.5 mm was measured per well.

### RNA extraction and real time quantitative PCR

Total RNA was extracted using RNeasy Mini Kit (Qiagen) to analyze gene expression using real time quantitative PCR (RT-qPCR). Briefly, cDNA was synthetized using GoScript reverse-transcription system (Promega). RT-qPCR was performed in triplicates with target-specific primers using a dye-based detection with GoTaq PCR master mix (Promega) or TaqMan Gene Expression Assay (Applied Biosystems) on AB7500 FAST real-time PCR system (Applied Biosystems). Expression levels were normalized to 3 internal controls tested for expression stability across samples in each experiment using Expression Suite Software (Life Technologies). Relative expression was calculated by 2−ΔΔCT method. (See supplemental Table S3 for list of primers and primer efficiency). For relative gene expression of the pericyte-like cells, normal human lung fibroblasts (CCD-16Lu) purchased from ATCC (CCL-204™) was set to one. DNA was extracted from passage 5 human lung fibroblasts and profiling of the cell line to determine its authenticity was performed using highly-polymorphic short tandem repeat loci (STRs) (Microsynth, Balgach, Switzerland).

### Statistical analysis

Data are expressed as mean ± SD. Comparisons between two groups were carried out using the parametric student’s two-tailed paired or unpaired t-test for normally distributed data. If data were not distributed normally, a nonparametric Wilcoxon signed-rank test was used between the two groups. One-way analysis of variance (ANOVA) followed by Newman-Keuls post hoc test was used for analysis of more than two groups. The numbers of samples (biological replicates) per group (n), or the numbers of experiments (technical replicates) are specified in the figure legends. Data was analyzed using GraphPad Prism 6 software. Statistical significance is accepted at p < 0.05.

## Electronic supplementary material

Movie 1

Movie 2

Movie 3

Supplementary information
