# Supplementary material for: Increased PD-L1 expression and IL-6 secretion characterize human lung tumor-derived perivascular-like cells that promote vascular leakage in a perfusable microvasculature model
Source: Sci Rep. 2017 Sep 6;7:10636. doi: 10.1038/s41598-017-09928-1 (PMC5587684; doi:10.1038/s41598-017-09928-1)
Supplement: Supplementary file 4 — Supplementary information [file 41598_2017_9928_MOESM4_ESM.pdf]

Supplementary Information for the manuscript

Increased PD-L1 expression and IL-6 secretion characterize human lung tumor-derived perivascular-like cells that promote vascular leakage in a perfusable microvasculature model

Colette A. Bichsel, Limei Wang, Laurène Froment, Sabina Berezowska, Stefan Müller, Patrick Dorn, Thomas M. Marti, Ren-Wang Peng, Thomas Geiser, Ralph A. Schmid, Olivier T. Guenat and Sean R.R. Hall

Supplementary Figures

Figure S1

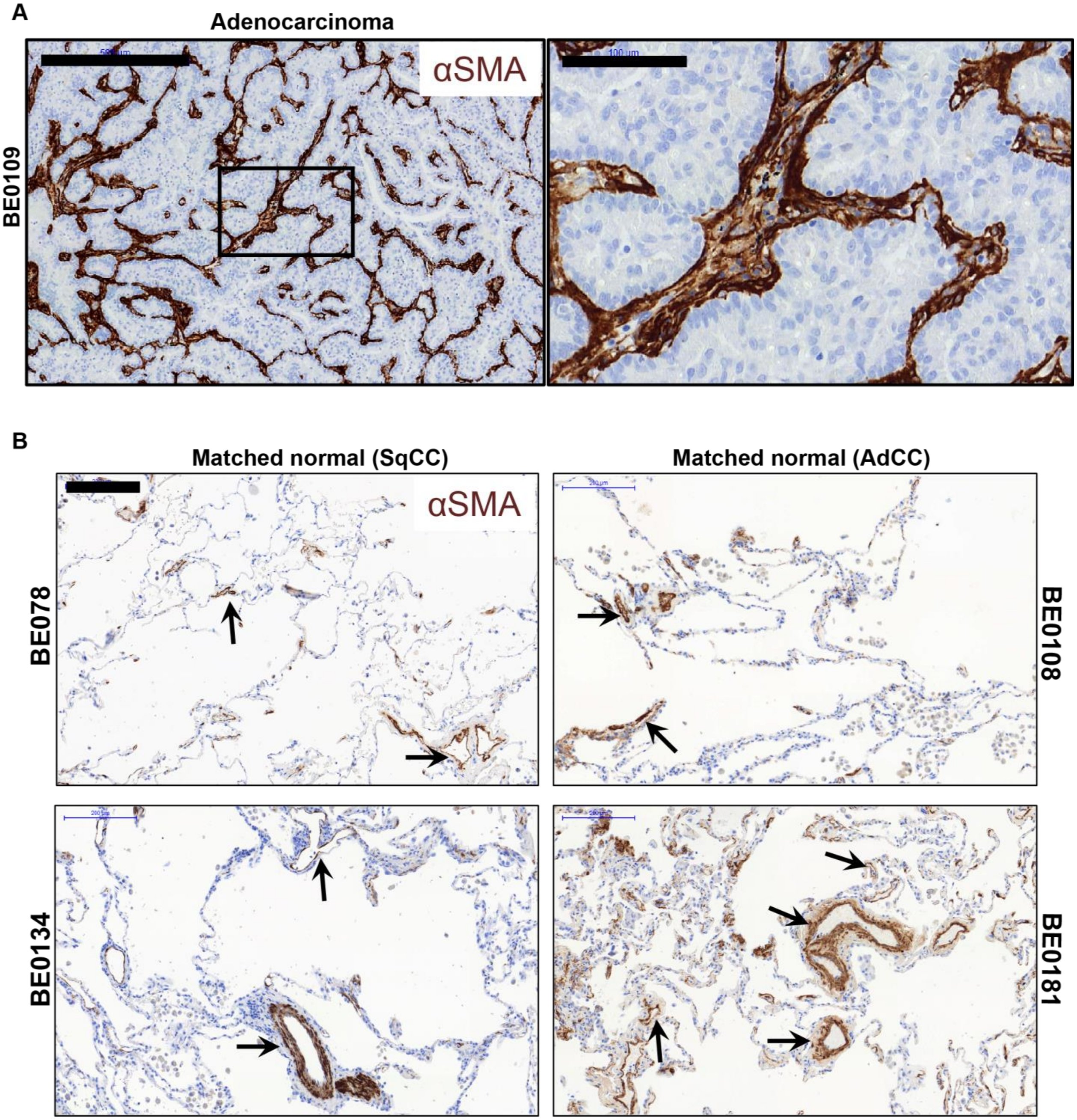

Figure S1. Presence of activated aSMA+ stroma in NSCLC. (A) Representative immunohistochemistry section from a lung adenocarcinoma patient specimen show aSMA+ stroma throughout the tumor foci. Scale bar 500 µm. Higher magnification in highlighted region (black rectangle). Scale bar 100 µm. (B) Representative sections taken from nonadjacent normal section of the lung from matched specimens. In the

distal area of the uninvolved normal lung, areas of aSMA can be seen primarily surrounding both small and large vessels (black arrows). Scale bar 200 μm. Related to Figure 1.

Figure S2

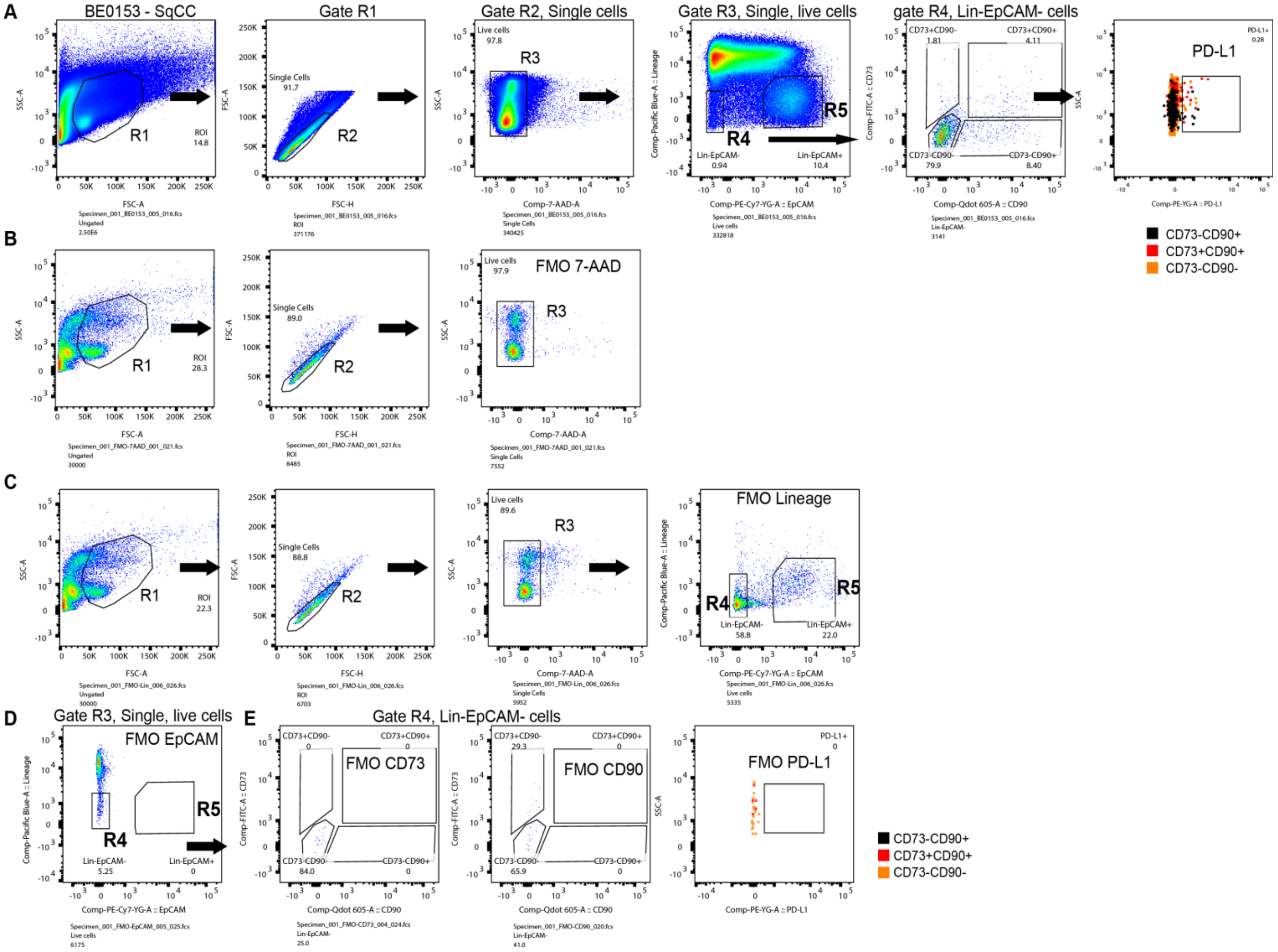

Figure S2. Fluorescence minus one controls to identify mesenchymal cell subsets in NSCLC. Representative experiment showing the sequential gating strategy and fluorescence minus one (FMO) controls to properly set cell population boundaries. (A) Sequential gating strategy demonstrating detection of mesenchymal cell subsets that express PD-L1 in a representative NSCLC specimen (BE0153-SqCC). (B) R1 gate initially displayed on a SSC/FSC color density plot subgated to select single cells based

on FSC-A versus FSC-H (R2 gate), which was further subgated for identification of live (FMO 7-AAD) cells (R3 gate). (C) R3 gate identifying single, live cells subgated to show the boundary of Lin+ population (FMO Lineage) from the mesenchymal (gate R4, Lin-EpCAM-) and epithelial (gate R5, Lin-EpCAM+) populations. (D) Single, live cells (Gate R3) were displayed on a bivariate plot to determine the boundary of the Lin-EpCAM- (gate R4) population using FMO EpCAM. (E) Lin-EpCAM- cells (Gate R4) displayed as a bivariate plot to identify the boundary for CD73+ cells (left, FMO CD73), CD90+ cells (centre, FMO CD90) and PD-L1 (FMO PD-L1). Related to Figure 2.

Figure S3

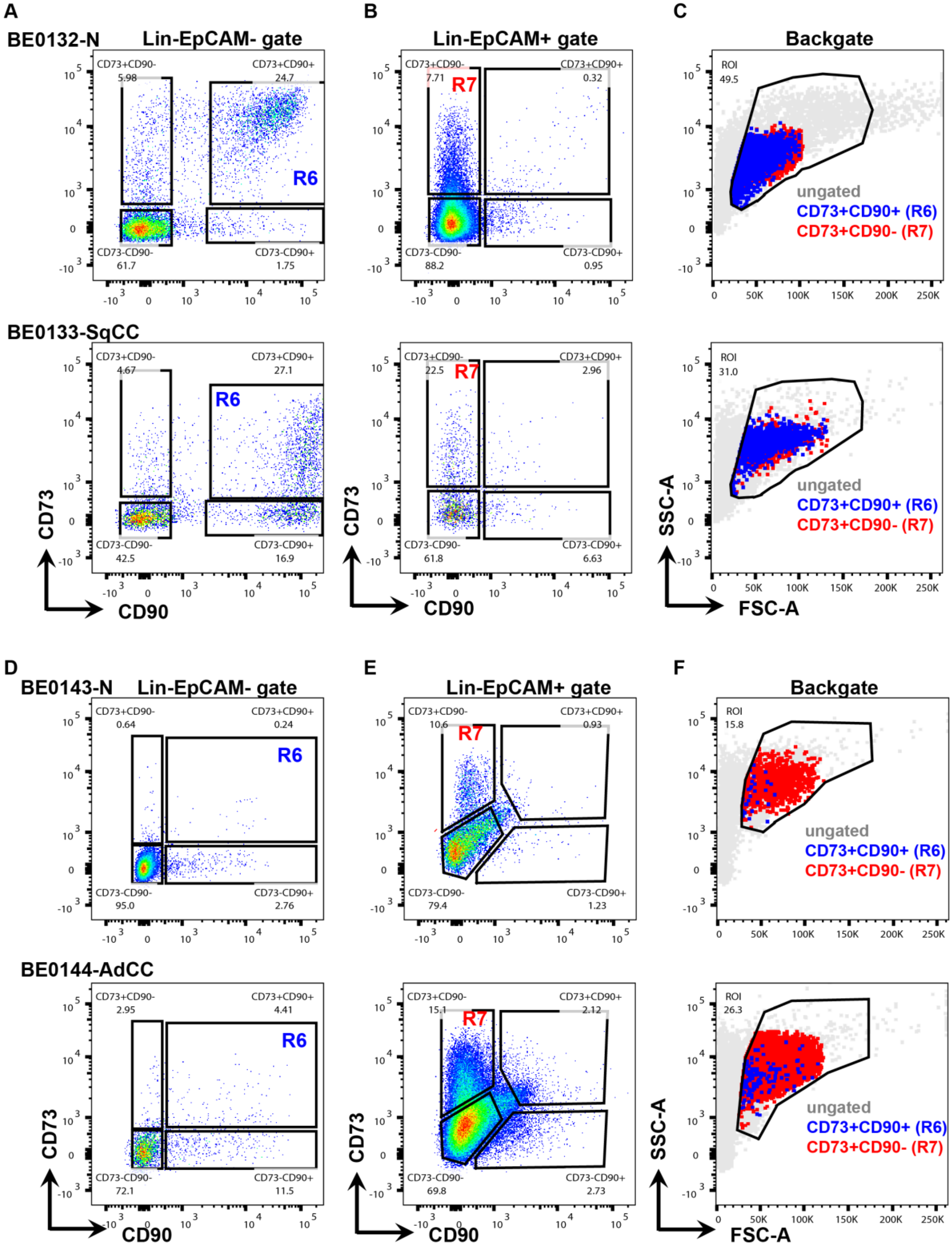
A
BE0132-N
Lin-EpCAM- gate
CD73+CD90- 5.98
CD73+CD90+ 24.7
R6
CD73-CD90- 61.7
CD73-CD90+ 1.75
B
Lin-EpCAM+ gate
CD73+CD90- 7.71
R7
CD73+CD90+ 0.32
CD73-CD90- 88.2
CD73-CD90+ 0.95
C
Backgate
ROI 49.5
ungated
CD73+CD90+ (R6)
CD73+CD90- (R7)
BE0133-SqCC
CD73+CD90- 4.67
CD73+CD90+ 27.1
R6
CD73-CD90- 42.5
CD73-CD90+ 16.9
CD73+CD90- 22.5
R7
CD73+CD90+ 2.96
CD73-CD90- 61.8
CD73-CD90+ 6.63
ROI 31.0
ungated
CD73+CD90+ (R6)
CD73+CD90- (R7)
CD73
CD90
SSC-A
FSC-A
D
BE0143-N
Lin-EpCAM- gate
CD73+CD90- 0.64
CD73+CD90+ 0.24
R6
CD73-CD90- 95.0
CD73-CD90+ 2.76
E
Lin-EpCAM+ gate
CD73+CD90- 10.6
R7
CD73+CD90+ 0.93
CD73-CD90- 79.4
CD73-CD90+ 1.23
F
Backgate
ROI 15.8
ungated
CD73+CD90+ (R6)
CD73+CD90- (R7)
BE0144-AdCC
CD73+CD90- 2.95
CD73+CD90+ 4.41
R6
CD73-CD90- 72.1
CD73-CD90+ 11.5
CD73+CD90- 15.1
R7
CD73+CD90+ 2.12
CD73-CD90- 69.8
CD73-CD90+ 2.73
ROI 26.3
ungated
CD73+CD90+ (R6)
CD73+CD90- (R7)
CD73
CD90
SSC-A
FSC-A

Figure S3. Back-gating strategy shows location of mesenchymal cell subsets in NSCLC. Backgating strategy to visualize final gated cells on previous plots in representative NSCLC samples and matched nonadjacent normal tissue. Bivariate plots identifying clusters of mesenchymal (A) and epithelial cells (B) in normal (BE0132-N, upper plots) and NSCLC specimen (BE0133-SqCC, lower plots). R6 and R7 gates displayed on a SSC/FSC color density plot to display their location using backgating. (D-F) Representative plots showing backgating strategy showing location of cells in gates R6 and R7 for a Adenocarcinoma specimen (BE0144-Ad). Related to Figure 2.

Figure S4

A

Lin-EpCAM- gate R4

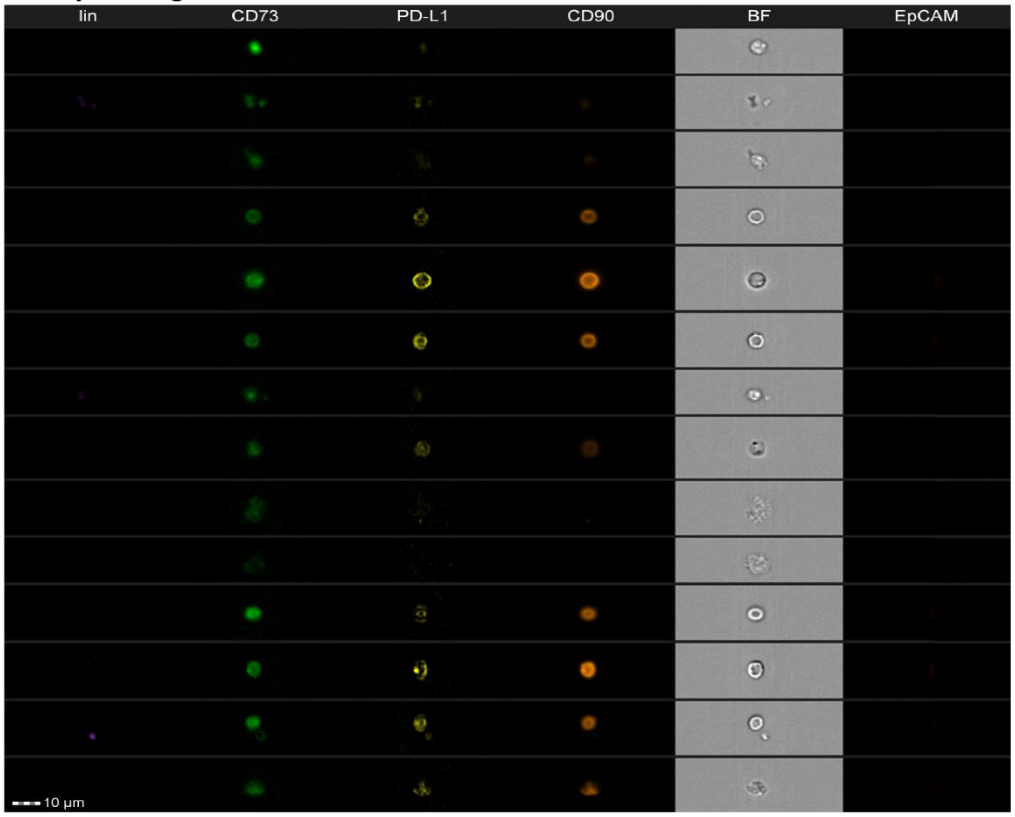

B

Lin-EpCAM+ gate R5

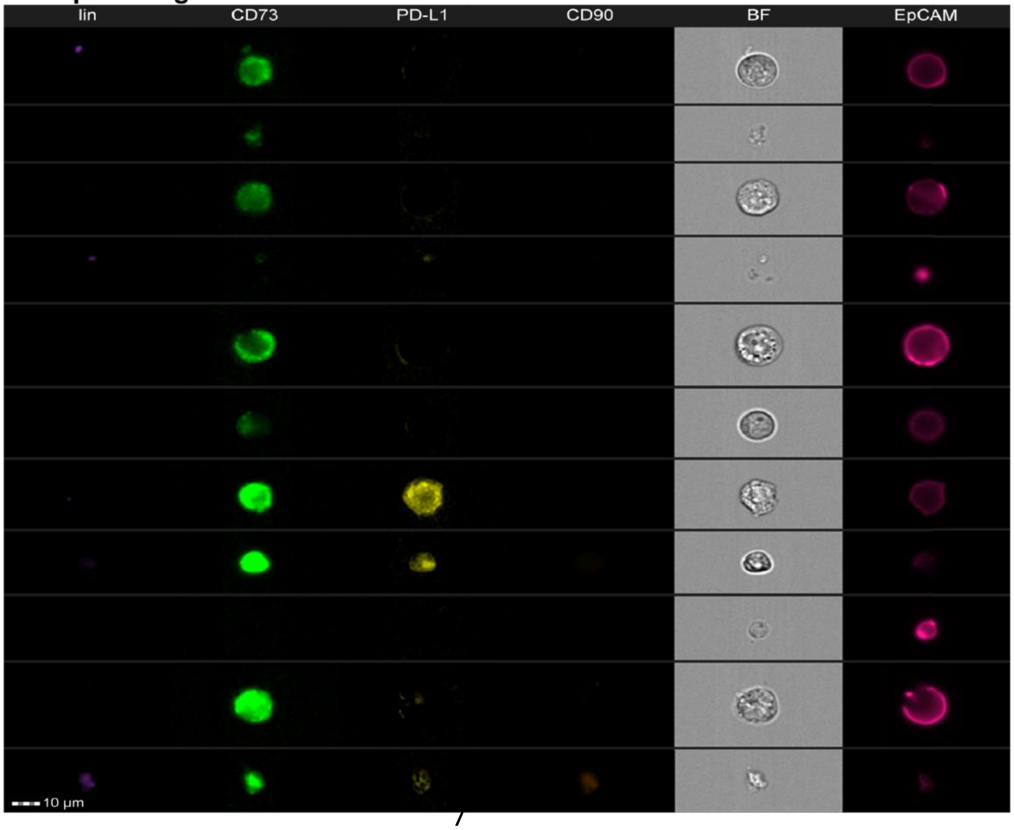

Figure S4. Tumor-derived perivascular-like cells. (A-B) Representative images of single cells obtained from a lung adenocarcinoma patient using the ImageStream® system (Amnis, Millipore). Each cell is represented by six images in a row acquired simultaneously showing with high sensitivity both morphology via transmitted light (BF, bright field) and membranous fluorescent staining pattern. Related to Figure 2E and I.

Figure S5

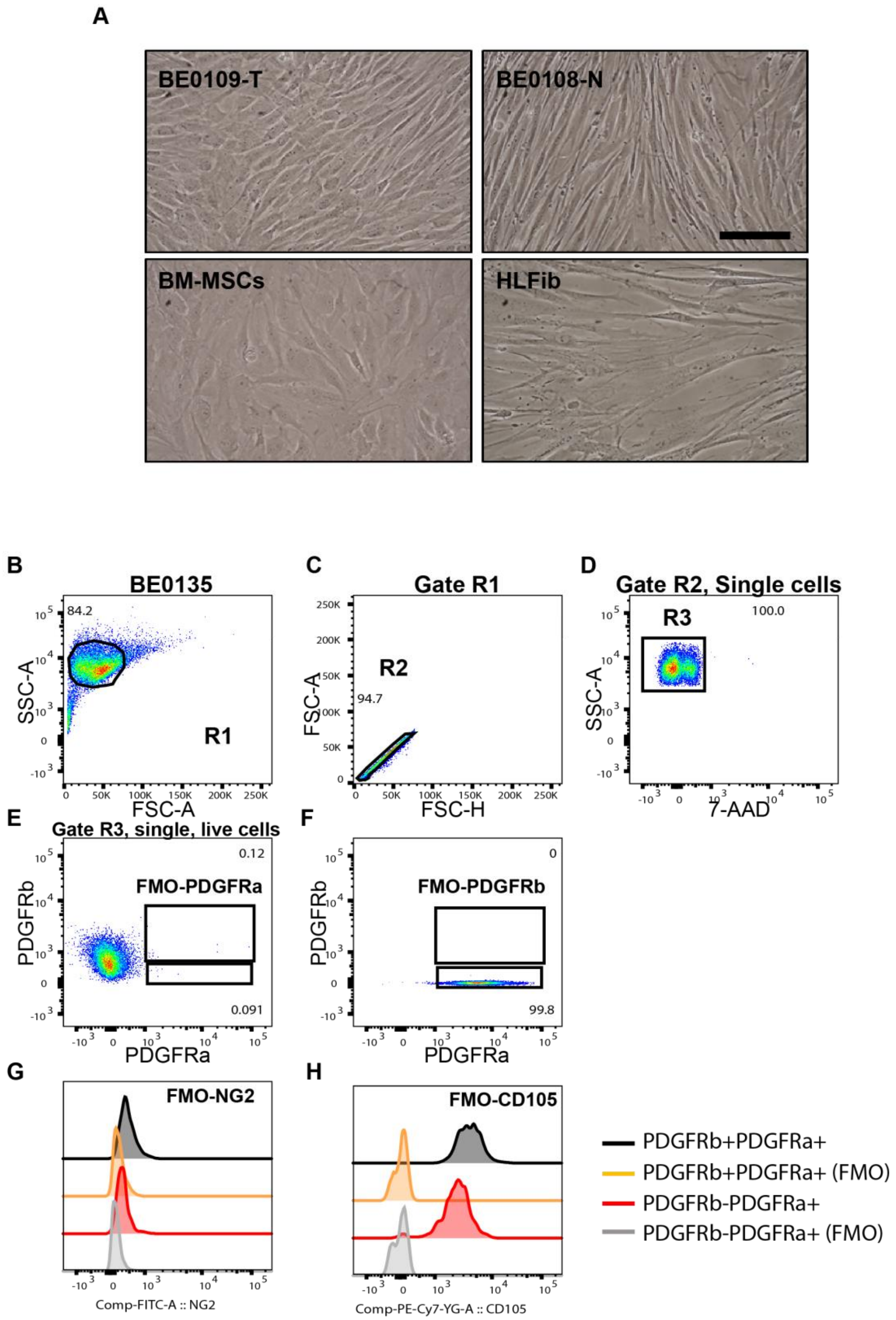

A
BE0109-T
BE0108-N
BM-MSCs
HLFib
B
BE0135
84.2
SSC-A
FSC-A
R1
C
Gate R1
R2
94.7
FSC-A
FSC-H
D
Gate R2, Single cells
R3
100.0
SSC-A
7-AAD
E
Gate R3, single, live cells
0.12
FMO-PDGFRa
PDGFRb
PDGFRa
0.091
F
0
FMO-PDGFRb
PDGFRb
PDGFRa
99.8
G
FMO-NG2
Comp-FITC-A :: NG2
H
FMO-CD105
Comp-PE-Cy7-YG-A :: CD105
PDGFRb+PDGFRa+
PDGFRb+PDGFRa+ (FMO)
PDGFRb-PDGFRa+
PDGFRb-PDGFRa+ (FMO)

Figure S5. (A) Representative phase contrast images of tumor and normal Lin-EpCAM-CD73+CD90+ cells, as well as human lung fibroblasts and BM-MSCs. Scale bar 50µm. (B-G) FMO strategy to show location of boundaries for various immunophenotypic markers in culture expanded cells. Related to Figure 3.

Figue S6

A

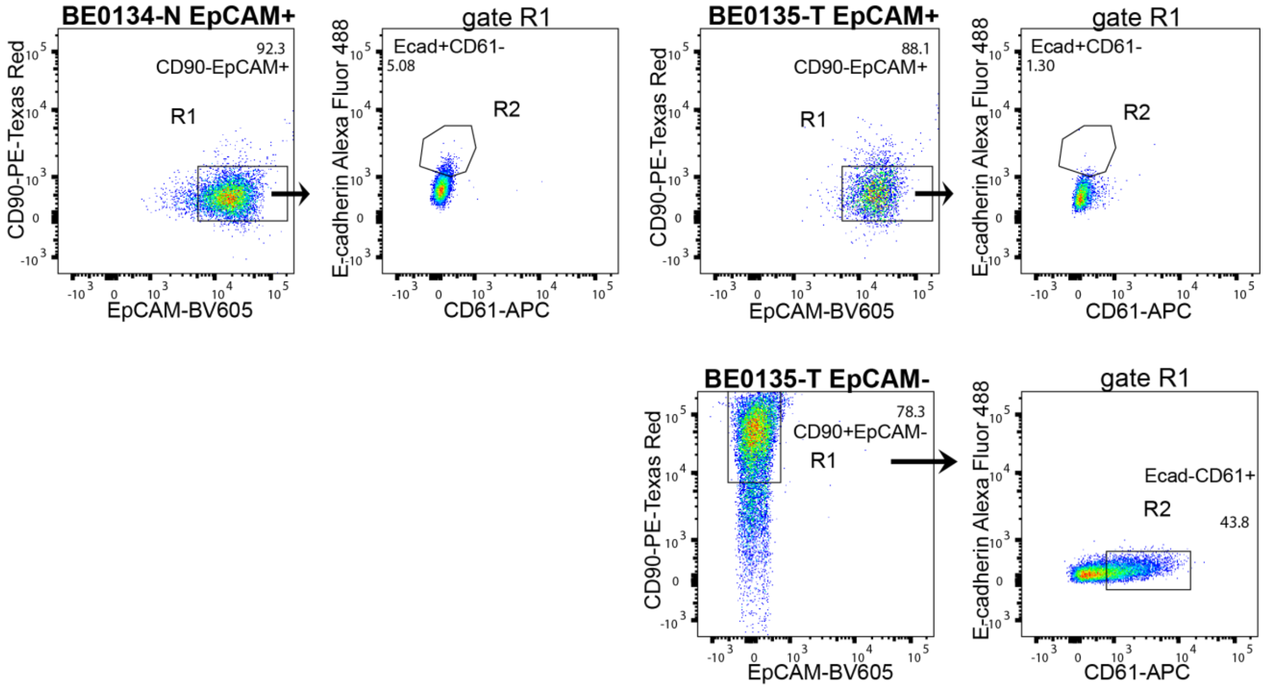

BE0134-N EpCAM+
92.3
CD90-EpCAM+
R1
CD90-PE-Texas Red
EpCAM-BV605
gate R1
Ecad+CD61-
5.08
R2
E-cadherin Alexa Fluor 488
CD61-APC
BE0135-T EpCAM+
88.1
CD90-EpCAM+
R1
gate R1
Ecad+CD61-
1.30
R2
BE0135-T EpCAM-
78.3
CD90+EpCAM-
R1
gate R1
Ecad-CD61+
R2
43.8

B

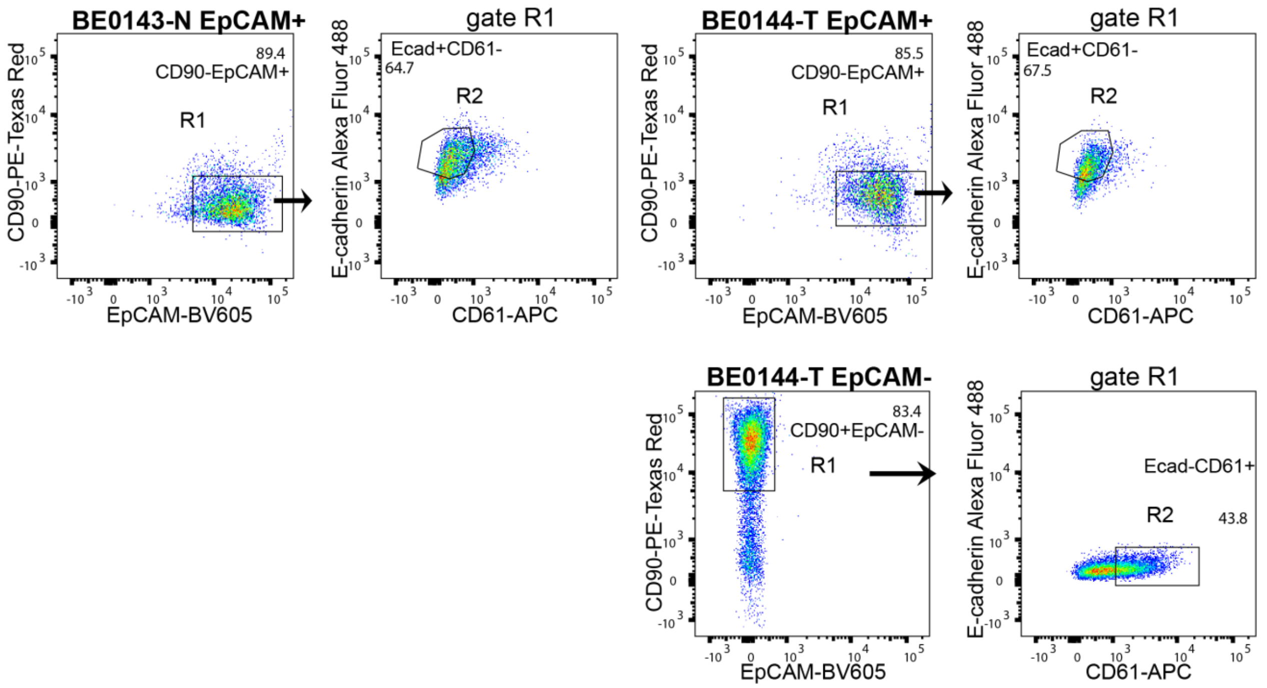

BE0143-N EpCAM+
89.4
CD90-EpCAM+
R1
CD90-PE-Texas Red
EpCAM-BV605
gate R1
Ecad+CD61-
64.7
R2
E-cadherin Alexa Fluor 488
CD61-APC
BE0144-T EpCAM+
85.5
CD90-EpCAM+
R1
gate R1
Ecad+CD61-
67.5
R2
BE0144-T EpCAM-
83.4
CD90+EpCAM-
R1
gate R1
Ecad-CD61+
R2
43.8

Figure S6. (A) Representative bivariate flow cytometric plots showing the expression of CD90-PE-Texas Red, EpCAM-BV605™, E-Cadherin Alexa Fluor®488 and CD61-APC in prospectively isolated, single, live Lin-EpCAM-CD73+CD90+ and Lin-EpCAM+CD73+CD90- cells following their expansion from tumor and matched normal specimens. BE0134 (normal) and BE0135 (tumor) (A) and BE0143 (normal) and BE0144 (tumor) (B) are shown. FMO strategy was used to determine the location of boundaries for the immunophenotypic markers in culture expanded cells (data not shown).

Figure S7

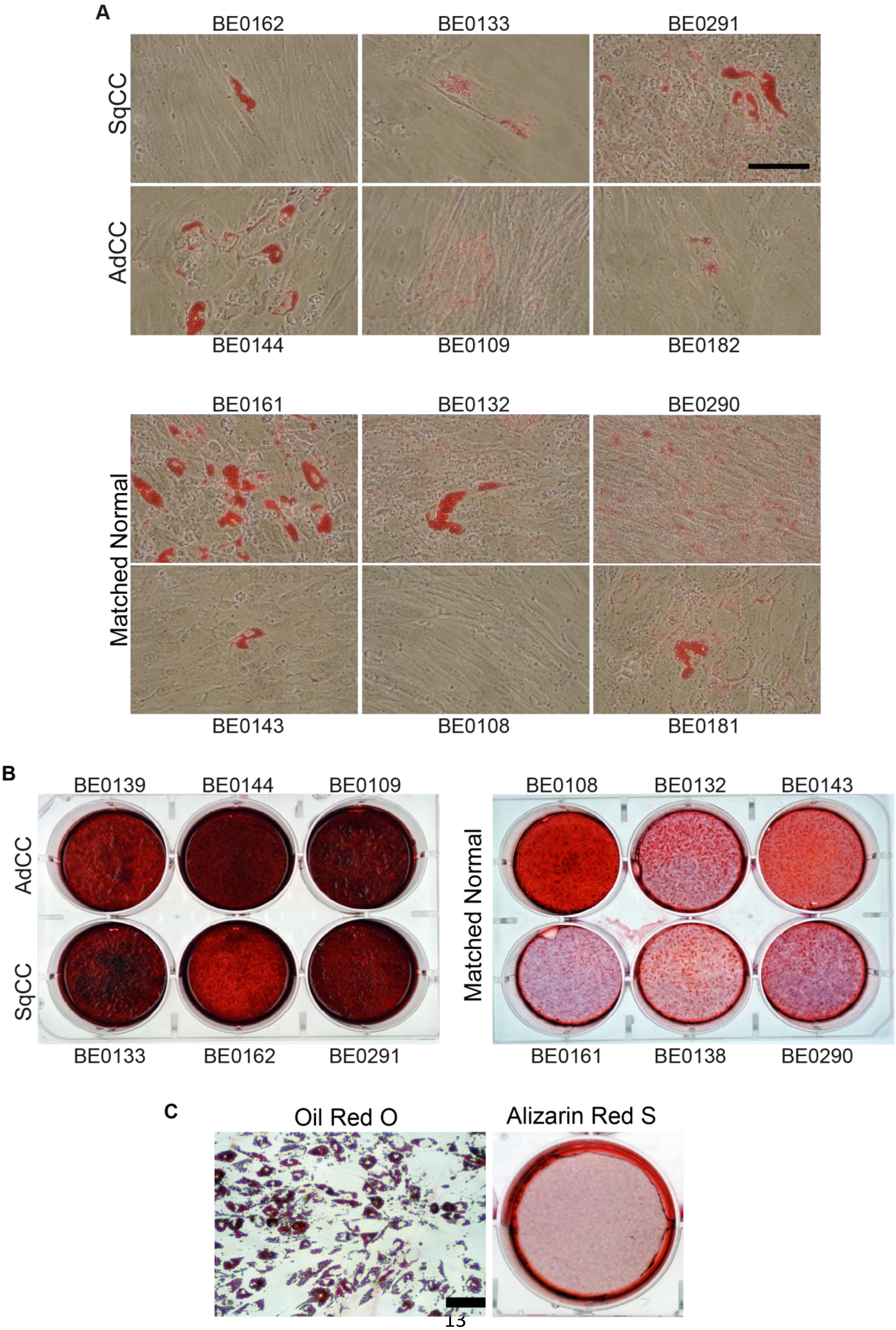
A
BE0162
BE0133
BE0291
SqCC
AdCC
BE0144
BE0109
BE0182
BE0161
BE0132
BE0290
Matched Normal
BE0143
BE0108
BE0181
B
BE0139
BE0144
BE0109
AdCC
SqCC
BE0133
BE0162
BE0291
BE0108
BE0132
BE0143
Matched Normal
BE0161
BE0138
BE0290
C
Oil Red O
Alizarin Red S

Figure S7. Differentiation potential of Lin-EpCAM-CD73+CD90+ perivascular cells. and bone marrow-derived mesenchymal stromal cells isolated from a healthy donor. (A) Phase contrast image showing uptake of Oil Red O in tumor (upper panels) and matched normal (lower panels) Lin-EpCAM-CD73+CD90+ perivascular cells. Scale bar 50 µm. (B) Alizarin Red S staining in tumor (left panels) and matched normal (right panels) Lin-EpCAM-CD73+CD90+ perivascular cells. (C) Representative image of BM-adipogenic differentiation in BM-MSCs depicted by uptake of Oil Red O (left panel). Osteogenic differentiation potential in BM-MSCs depicted by Alizarin Red S staining (right panel). Scale bar 50 µm.

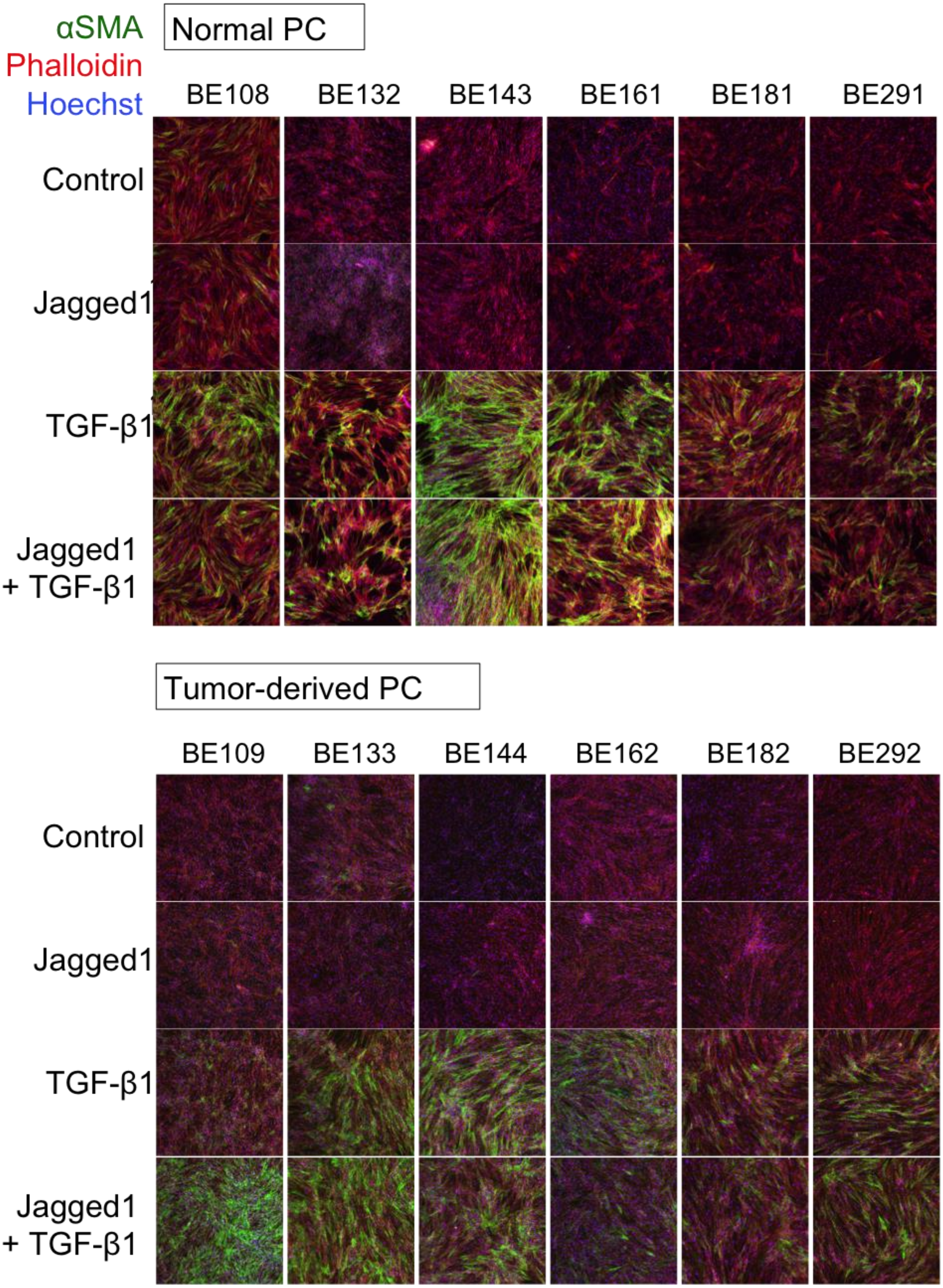

αSMA
Phalloidin
Hoechst
Normal PC
BE108
BE132
BE143
BE161
BE181
BE291
Control
Jagged1
TGF-β1
Jagged1 + TGF-β1
Tumor-derived PC
BE109
BE133
BE144
BE162
BE182
BE292
Control
Jagged1
TGF-β1
Jagged1 + TGF-β1

Figure S8. Upregulation of αSMA in tumor-derived Lin-EpCAM-CD73+CD90+ mesenchymal cells and their matched normal counterpart. Representative images taken from individual matched specimens three days after treatment with TGF-β1, Jagged1 or untreated control showing changes in phalloidin (red) and αSMA (green). Related to Figure 4.

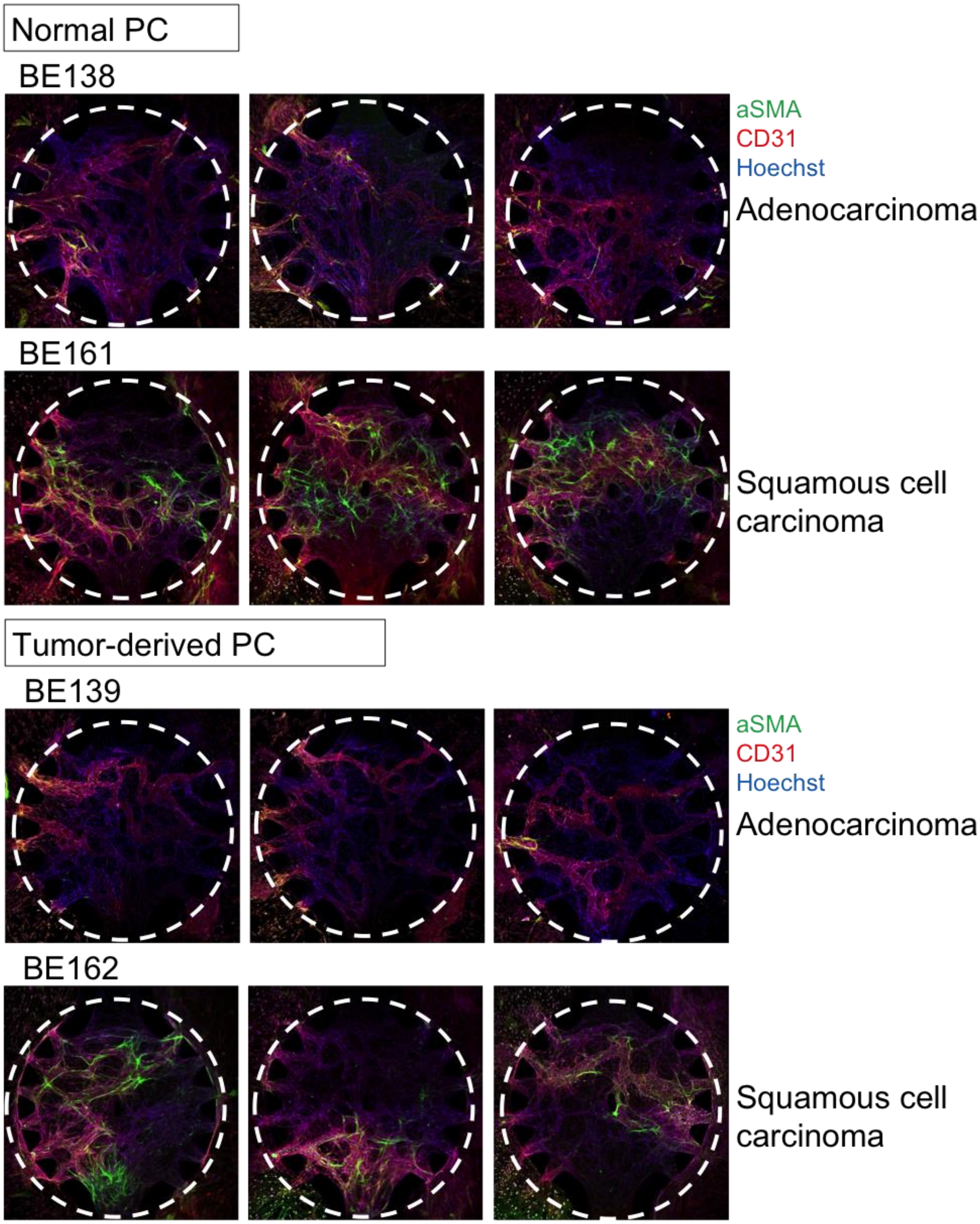

Figure S9. Whole-chip images (central chamber of 2.4mm delineated with a dashed line) with αSMA and CD31 staining reveal a very heterogeneous upregulation in tumor-derived pericytes around endothelial vessels. Related to Figure 5.

Table S1. Patient Characteristics

Patient ID	Histological type	Sex	DOB	Smoker	Grade	Stage	Treatment
BE063	Adenocarcinoma	f	03.11.1941	not reported	G3	IIB	none
BE079	Squamous cell carcinoma	f	10.01.1941	Y	G3	IIIA	none
BE0109	Adenocarcinoma	m	13.07.1953	y	G2	IA	none
BE0133	Squamous cell carcinoma	m	09.03.1950	y	G2	IA	none
BE0135	Squamous cell carcinoma	m	12.09.1950	y	G2	IV	none
BE0139	Adenocarcinoma	f	19.07.1956	y	G2	IIIA	none
BE0144	Adenocarcinoma	m	29.08.1963	no	nr	IIIA	Cisplatin
BE0153	Squamous cell carcinoma	f	02.07.1942	former	G2	IIB	none
BE0162	Squamous cell carcinoma	f	02.03.1940	y	G3	IIIA	none
BE0182	Adenocarcinoma	m	11.08.1943	not reported	G2	IB	none
BE0190	Adenocarcinoma	m	03.03.1931	former	G2	IA	none
BE0242	Squamous cell carcinoma	m	25.02.1938	y	G2	IB	none
BE0291	Squamous cell carcinoma	m	28.05.1946	y	nr	IIA	Cisplatin/Taxotere

y, yes

Table S2. List of antibodies used for FACS/flow cytometric analysis

Primary Antibodies	Manufacturer	Clone	Catalog #
CD326 (EpCAM)-PE-Cy7	eBioscience	1B7	25-9326-42
CD326 (EpCAM)-BV605™	BioLegend	9C4	324224
CD73-FITC	eBioscience	AD2	11-0739-42
CD73-APC	eBioscience	AD2	17-0739-42
CD90-BV605	BioLegend	5E10	328128
CD90-PE-TR	BD Horizon™	5E10	562385
CD45-eFluor®450	eBioscience	2D1	48-9459-42
CD14-eFluor®450	eBioscience	61D3	48-0149-42
CD31-eFluor®450	eBioscience	WM-59	48-0319-42
CD235a (GlyA)-eFluor®450	eBioscience	HIR2	48-9987-42
CD274 (PD-L1)-FITC	BD Biosciences	MIH1	558065
CD274 (PD-L1)-PE	BioLegend	29E.2A3	329706
CD105-PE-Cy7	BioLegend	14G2a	357306
CD146-FITC	eBioscience	P1H12	11-1469-42
CXCR4-BV711	BD Biosciences	12G5	740799
GD2-APC	BioLegend	14G2a	357306
NG2-FITC	eBioscience	9.2.27	53-6504-42
CD54-Pacific Blue™	BioLegend	HA58	353110
PDGFRα-PE	BioLegend	16A1	323506
PDGFRβ-APC	BioLegend	18A2	323608
CD61-APC	eBioscience	V1-PL2	17-0619-42
E-Cadherin Alexa Fluor® 488	BD Pharmingen™	36/E-Cadherin	560061
7-AAD	eBioscience		00-6990-50

Table S3. Dye-based detection primers

Target Gene	Forward Primer (5‘-3‘)	Reverse Primer (5‘-3‘)	*RefSeq ID*	*Efficiency*
POSTN	CAACGGGCAAATACTGGAAAC	TCTCGCGGAATATGTGAATCG	*NM_001135934.1*	*90%*
TP53	GCTTTCCACGACGGTGAC	GCTCGACGCTAGGATCTGAC	*NM_000546*	*100%*
FAP	ATCTATGACCTTAGCAATGGAGAATTTGT	GTTTGATAGACATATGCTAATTTACTCCCAAC	*NM_004460.3*	*93%*
GLI1	CCAGCGCCCAGACAGAG	GGCTCGCCATAGCTACTGAT	*NM_005269.2*	*98%*
RGS5	CCTCCTTCTGTTGCTCTCTTT	CAGTCACATAGTTGCTTCACTTTC	*NM_001195303.2*	*94%*
DES	CCAAGCAGGAGATGATGGAATA	CCTCATCAGGGAATCGTTAGTG	*NM_001927.3*	*97%*
CNN1	GTTTGAGAACACCAACCATACAC	CACGTTCACCTTGTTTCCTTTC	*NM_001299.5*	*93%*
ACTA2	GATGGTGGGAATGGGACAAA	GCCATGTTCTATCGGGTACTTC	*NM_001141945.1*	*99%*
VEGFa	CGA GGG CCT GGA GTG TGT	CGC ATA ATC TGC ATG GTG ATG	*NM_003376.5*	*99%*
**Internal control**				
TBP	AACAACAGCCTGCCACCTTA	GCCATAAGGCATCATTGGAC	*NM_003194*	*91%*
GAPDH	TCG ACA GTC AGC CGC ATC TTC TTT	GCC CAA TAC GAC CAA ATC CGT TGA	*NM_002046.5*	*98%*
H3F3A	GGT GTC TTC AAA AAG GCC AA	GCG AGA AAT TGC TCA GGA CT	*NM_002107*	*98%*

TaqMan primer/probe assay

Target Gene	Assay ID	*RefSeq ID*
PD-L1	Hs00204257_m1	*NM_001314029.1*
**Internal control**		
TBP	Hs00427620_m1	*NM_003194.4*
GADPH	Hs02758991_g1	*NM_002046.5*
H3F3A	Hs02598544_g1	*NM_002107.4*

Supplementary movies

Supplementary movie S1. 3D rendering of tumor pericyte-endothelial cell interaction in patent formed vascular structure in microfluidic chip.

Supplementary movie S2. Time-lapse video of RITC-dextran perfusion through a microvascular network stabilized by normal pericytes.

Supplementary movie S3. Time-lapse video of RITC-dextran perfusion through a microvascular network stabilized by tumor-derived pericytes.
